# UHRF1 Controls the Timing of RAD51 Removal During DNA Damage Repair Through Suppressing RFWD3

**DOI:** 10.1002/advs.202509901

**Published:** 2025-09-12

**Authors:** Guangxue Liu, Kaiyan Huang, Shiyao Liu, Ziyu Sheng, Pumin Zhang

**Affiliations:** ^1^ Zhejiang Provincial Key Laboratory of Pancreatic Disease The First Affiliated Hospital of Zhejiang University Hangzhou Zhejiang 310003 China; ^2^ Institute of Translational Medicine Zhejiang University School of Medicine Hangzhou Zhejiang 310058 China; ^3^ Cancer Center Zhejiang University Hangzhou Zhejiang 310058 China

**Keywords:** DNA damage, RAD51, RFWD3, UHRF1

## Abstract

The RAD51 recombinase is evolutionarily conserved critical for homologous recombination (HR)‐mediated repair of DNA double‐strand breaks. It binds to single strand DNA to form protein‐DNA filaments for homology searching and pairing during HR repair. RFWD3 is an E3 ubiquitin ligase shown to remove RAD51 at the completion of HR repair through ubiquitination and degradation of RAD51. However, it remains elusive what prevents RFWD3 from attacking RAD51 in the absence of DNA damage and early on during the repair process. Here, we show that it is UHRF1 that protects RAD51, and it does so by acting as an E3 ubiquitin ligase of RFWD3 is demonstrated. Interestingly, RAD51 also protects RFWD3 from UHRF1, thereby establishing a negative feedback circuit that regulates the protein levels of RFWD3 and RAD51. Furthermore, it is shown that the ubiquitination of RFWD3 is regulated by phosphorylation status of UHRF1, and that phosphatase PP4 is important for modulating UHRF1 activity. Altogether, these regulatory mechanisms ensure that the recombinase RAD51 is maintained at appropriate levels for HR repair.

## Graphical abstract

We demonstrate here that the recombinase RAD51 is protected by UHRF1 through ubiquitinating RFWD3, an E3 for RAD51, and this very process is inhibited by RAD51, leading to a triple negative feedback circuit that ensures appropriate levels of RFWD3 and RAD51 for DNA damage response.

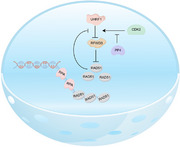

## 1 Introduction

UHRF1 (ubiquitin‐like with PHD and ring finger domains 1) is best known for its role in the maintenance methylation of genomic DNA.^[^ ^1^, ^2^ ^]^ It contains an array of functional domains including (from N‐terminus to C‐terminus): a ubiquitin‐like (UBL) domain, a tandem Tudor domain (TTD), a plant homeodomain (PHD), a SET‐ and RING‐associated (SRA) domain, and a RING‐finger domain. The TTD and PHD domains are histone readers with the TTD recognizing the heterochromatin marker: histone H3 trimethylated at lysine 9 (H3K9me3) ^[^ ^3^, ^4^, ^5^, ^6^, ^7^, ^8^, ^9^, ^10^ ^]^ and the PHD binding to the extreme N‐terminus of H3 at arginine 2 (R2).^[^ ^11^, ^12^ ^]^ The SRA domain binds to hemi‐methylated CpG dinucleotides,^[^ ^13^, ^14^, ^15^ ^]^ and the RING‐finger domain contains E3 activity to ubiquitinate H3 ^[^ ^16^ ^]^ and many other substrates as well. The UBL domain helps organize these various functional modules and brings in E2 enzymes for the RING‐finger to function as an E3 ubiquitin ligase.^[^ ^17^ ^]^ These functional domains work together to mono‐ubiquitinate the K23 and K18 residues of histone H3 at hemi‐methylated regions of the genome during or after replication to generate DNMT1 recruitment signal.^[^ ^16^ ^]^ The recruited DNMT1 then methylates the nascent DNA strand.

It has been well established now that UHRF1 also plays important roles in DNA damage and replication stress responses.^[^ ^18^ ^]^ Its various domains not only play important functions in the maintenance methylation but also in DNA damage response. The SRA domain can recognize DNA lesions such as inter‐strand crosslink.^[^ ^19^, ^20^ ^]^ The TTD domain binds to H3K9me3 which is enriched in DNA damage sites,^[^ ^21^ ^]^ helping UHRF1 recruitment to damage sites. Once at the damage sites, its PHD domain may reinforce the recruitment through binding to H3R2. UHRF1 also interacts with a large number of DNA damage response proteins including BRCA1,^[^ ^22^ ^]^ XLF,^[^ ^23^ ^]^ PARP1,^[^ ^24^ ^]^ etc. Its interaction with BRCA1 impacts repair choice by driving RIF1 away from double strand breaks so that the homologous recombination‐dependent repair is favored.^[^ ^22^ ^]^

The RAD51 recombinase is a homologue of bacterial RecA protein and plays a critical role in the homologous recombination‐mediated repair of DNA double strand breaks.^[^ ^25^ ^]^ It is well established that RAD51 binds to single strand DNA (ssDNA) to form protein‐DNA filaments which are required for homology searching and pairing, an essential step of homologous recombination repair.^[^ ^26^ ^]^ Upon completion of this step, RAD51 is removed from DNA strands by an ubiquitin ligase, RFWD3 (ring finger and WD repeat domain 3), through ubiquitination and proteasomal degradation.^[^ ^27^ ^]^ However, it is entirely unclear what prevents RFWD3 from attacking RAD51 early on in the HR process and in the absence of DNA damage.

Maintaining the protein level of RAD51 is important as we recently identified a novel function of the recombinase in the maintenance methylation of genomic DNA by DNMT1.^[^ ^28^ ^]^ This function entails dual regulation of UHRF1. First, RAD51 directly inhibits UHRF1‐mediated poly‐ubiquitination and degradation of DNMT1, and second, RAD51 binds histone H3 and presents it to UHRF1 for ubiquitination to generate DNMT1 recruitment signal. Deficiency in *RAD51* causes excessive proteasomal degradation of DNMT1 and failed recruitment of the methyltransferase to the chromatin, resulting in the loss of global methylation of genomic DNA. Interestingly, we also found that RAD51 not only directly interacts and regulates UHRF1 but also depends on UHRF1 for its own expression.^[^ ^28^ ^]^ Here we report that UHRF1 maintains the stability of RAD51 by targeting RFWD3. Compromising *UHRF1* function leads to decreased proteasomal degradation of RFWD3 and thus increased expression levels of this ubiquitin ligase of RAD51, causing the recombinase to be excessively ubiquitinated and degraded. However, in a twist, RAD51, the very prey of RFWD3, actually protects RFWD3 from ubiquitination and destruction initiated by UHRF1. Thus, these three proteins form a negative feedback circuit that ensures proper expression levels of RFWD3 and RAD51 to function in DNA damage repair. Moreover, we show that UHRF1 is critical in protecting RAD51 from RFWD3 at damage sites, and provide evidence that phosphorylation status of UHRF1 also contributes to restraining RFWD3 from precociously degrading RAD51.

## 2 Results

### 2.1 The Stability of RAD51 Depends on UHRF1

**Figure 1:**
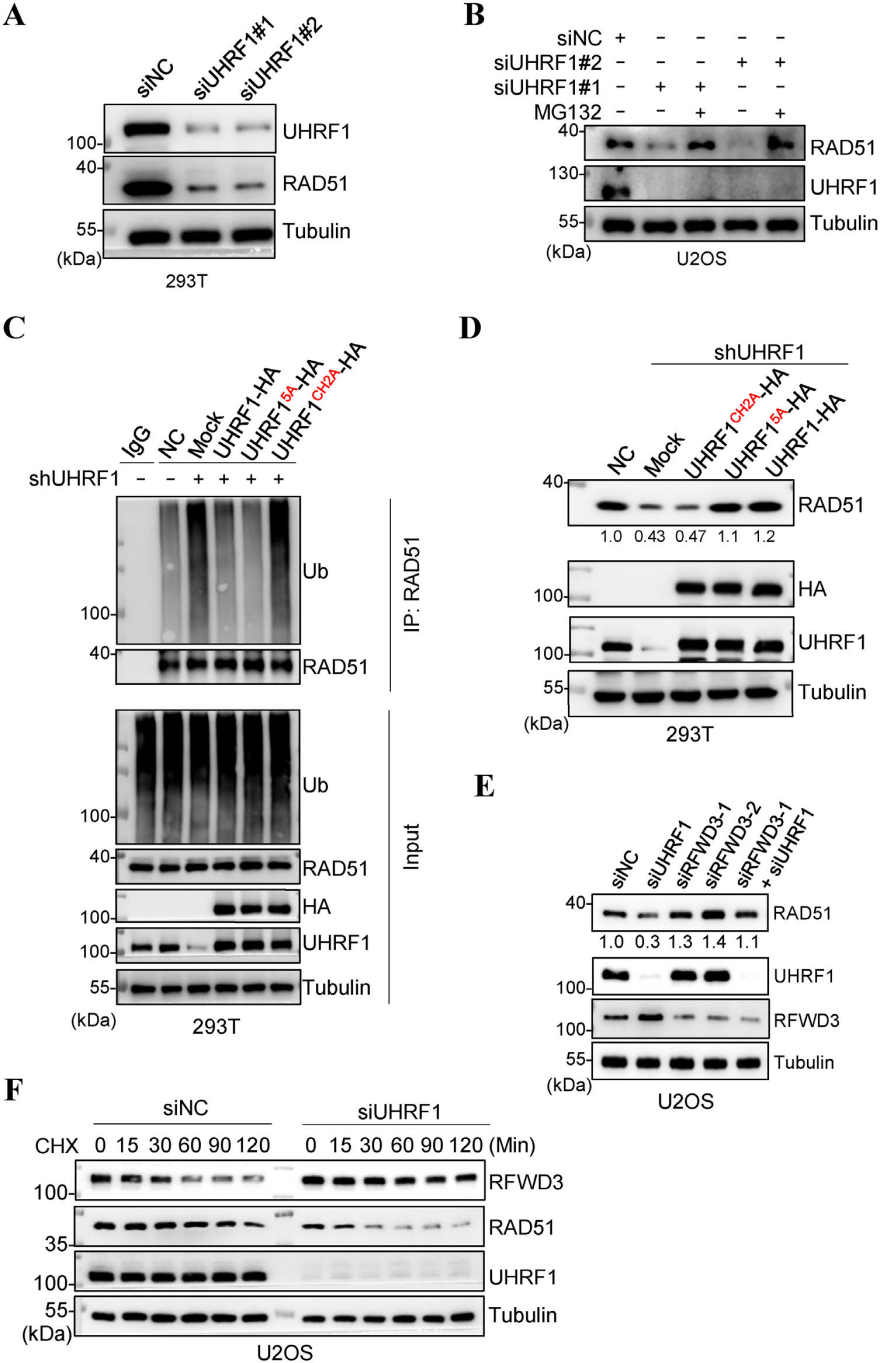
UHRF1 stabilizes RAD51 expression via suppressing RFWD3. A) Western blotting analysis of RAD51 protein levels following *UHRF1* knockdown in HEK293T cells. B) Western blotting analysis of RAD51 protein levels in *UHRF1*‐depleted U2OS cells. The cells were transfected with scrambled or *UHRF1* siRNA (two independent siRNAs) for 42 h, followed by 6 h treatment with 10 µm MG132. C) Ubiquitination assay of RAD51 in HEK293T cells depleted of *UHRF1* with re‐expression of *UHRF1*, *UHRF1^CH2A^* or *UHRF1^5A^*. D) Western blotting analysis of RAD51 protein levels in *UHRF1*‐depleted HEK293T cells with re‐expression of *UHRF1*, *UHRF1^CH2A^* or *UHRF1^5A^*. E) Western blotting analysis of RAD51 protein levels after knocking down *UHRF1* and *RFWD3*, individually or simultaneously in U2OS cells. F) Western blotting analysis of RAD51 and RFWD3 protein levels following *UHRF1* knockdown in U2OS cells. The cells were treated with cycloheximide (CHX, 50 µg mL^−1^) for the indicated times.

As we reported before,^[^ ^28^ ^]^ depleting the expression of *UHRF1* caused significant downregulation of RAD51 protein levels (**Figure** **1**A; Figure S1A, Supporting Information), but had no effect on the transcription (Figure S1A, Supporting Information). Such a downregulation could be prevented by suppressing the proteasome with MG132 in the *UHRF1‐*depleted cells (Figure 1B). On the other hand, the overexpression of *UHRF1* could increase the protein levels of RAD51 (Figure S1B, Supporting Information). These data suggest that RAD51 is ubiquitinated and degraded by the proteasome when *UHRF1* is compromised. Indeed, enhanced ubiquitination of RAD51 could be observed in *UHRF1*‐depleted cells (Figure 1C). To determine if the ubiquitin ligase activity of UHRF1 or the interaction with RAD51 ^[^ ^28^ ^]^ is required to maintain the stability of RAD51, we re‐expressed *UHRF1* in the depleted cells. Wildtype *UHRF1* could restore RAD51 levels, so could the RAD51 interaction‐deficient mutant (*UHRF1^5A^)*,^[^ ^28^ ^]^ indicating that UHRF1 stabilizes RAD51 without the need to interact with the recombinase (Figure 1D). However, the ubiquitin ligase‐dead mutant (*UHRF1^CH2A^*) ^[^ ^29^ ^]^ could not do the same (Figure 1D). In line with this, while both wildtype and the 5A mutant could prevent the excessive poly‐ubiquitination of RAD51, the CH2A mutant could not (Figure 1C).

Given the result that *UHRF1^CH2A^* could not stabilize RAD51, we suspected that UHRF1 targets another E3 ligase which can ubiquitinate RAD51 for degradation. When *UHRF1* is depleted, this E3 becomes upregulated and causes excessive degradation of RAD51. In addition to RFWD3,^[^ ^27^ ^]^ integrin β‐1‐suppressed RING1 was reported to be able to mediate RAD51 ubiquitination and degradation.^[^ ^30^ ^]^ Thus, RFWD3 and RING1 could be targeted by UHRF1. Alternatively, the reported RAD51 deubiquitinase UCHL3 ^[^ ^31^ ^]^ might require UHRF1 to sustain its expression somehow so that without *UHRF1*, its expression is downregulated and could not remove poly‐ubiquitin chains off RAD51, leaving the recombinase to be degraded. We therefore examined the levels of these proteins in *UHRF1‐*depleted cells. As shown in Figure S1C (Supporting Information), all but RFWD3 were not affected by *UHRF1* depletion. It was upregulated in the *UHRF1‐*depleted cells, suggesting that RFWD3 is the RAD51 E3 ligase targeted by UHRF1. Indeed, when the expression of *RFWD3* was depleted, RAD51 levels increased (Figure 1E), and overexpression of the E3 ligase suppressed RAD51 levels (Figure S1D, Supporting Information). Importantly, the downregulation of RAD51 caused by *UHRF1* depletion could be prevented in the cells depleted of both *UHRF1* and *RFWD3* (Figure 1E), indicating that UHRF1 stabilizes RAD51 through regulating RFWD3. Moreover, when *UHRF1* expression was depleted, RFWD3 became stabilized and at the same time, RAD51 became destabilized (Figure 1F).

To definitively rule out the possibility that UHRF1 mediates RAD51 ubiquitination to regulate the stability of the recombinase, we performed an in vitro ubiquitin assay with purified proteins. As shown in Figure S1E (Supporting Information), UHRF1 readily ubiquitinated itself, and such self‐ubiquitination was completely blocked by GST‐RAD51. However, no ubiquitination could be detected on GST‐RAD51. Thus, RAD51 binds UHRF1 and regulates its E3 activity against DNMT1 ^[^ ^28^ ^]^ and RFWD3 (see below), but itself is not a substrate of UHRF1.

### 2.2 UHRF1 Regulates RFWD3

**Figure 2:**
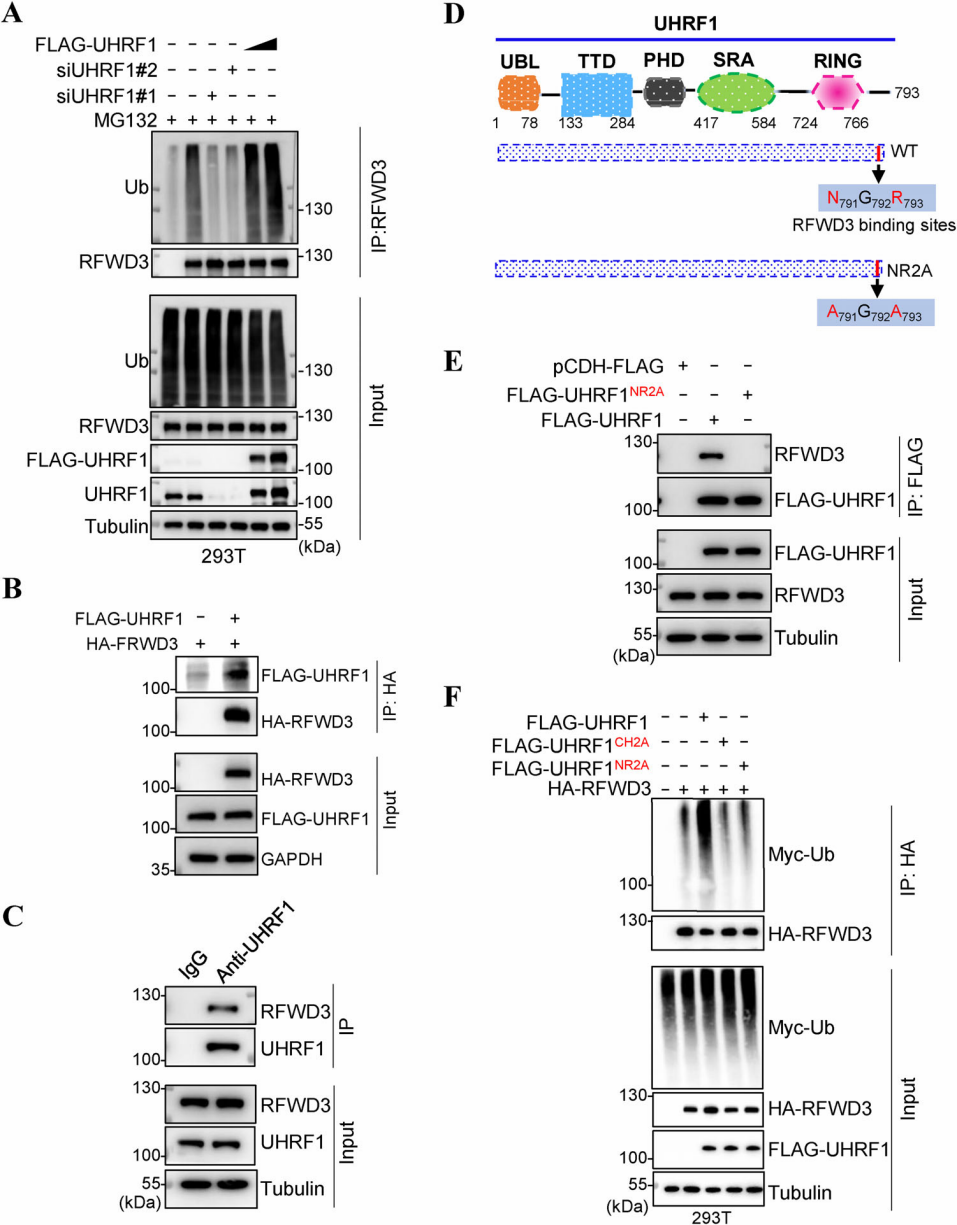
UHRF1 ubiquitinates RFWD3 for proteasomal degradation. A) Ubiquitination assay of RFWD3 in HEK293T cells with *UHRF1* depleted or overexpressed. B) Immunoprecipitation assay analysis of the ectopically expressed FLAG‐UHRF1 and RFWD3‐HA in HEK293T cells. C) Immunoprecipitation assay of endogenous UHRF1 and RFWD3 in HEK293T cells. D) Schematic drawing of UHRF1 illustrating the site that interacts with RFWD3. E) Immunoprecipitation assay of the ectopically expressed *RFWD3‐HA*, *FLAG‐UHRF1* and *FLAG‐UHRF1^NR2A^* in HEK293T cells. F) Ubiquitination assay of RFWD3 in HEK293T cells transfected with *RFWD3‐HA* together with *FLAG‐UHRF1*, *FLAG‐UHRF1^CH2A^* or *FLAG‐UHRF1^NR2A^*.

To demonstrate that UHRF1 is indeed an E3 for RFWD3, we performed a ubiquitination assay. As shown in **Figure** **2**A, the depletion of *UHRF1* diminished RFWD3 ubiquitination. Further, with ubiquitin mutants capable of only one type of K links, the ubiquitin modification on RFWD3 was shown to be K48‐linked (Figure S2A, Supporting Information). Immunoprecipitation assays also showed an interaction between UHRF1 and RFWD3 (Figure 2B,C), and the interaction is very likely a direct one as demonstrated by the GST pulldown assay (Figure S2B, Supporting Information). To determine the site in UHRF1 where RFWD3 binds to, we generated a series of truncated UHRF1 proteins, and found that once the C‐terminus was deleted, even as few as 13 residues, the interaction with RFWD3 was abolished (Figure S2B,C, Supporting Information). The last 3 residues of UHRF1 turned out to be the site that interacts with RFWD3. Mutating all 3 or 2 of the 3 residues, or even just the last one residue disrupts the interaction with RFWD3 (Figure 2D; Figure S2D, Supporting Information). To minimize the impact on UHRF1 function, we will be using the N791A/R793A mutant (*UHRF1^NR2A^*) for experimenting. This mutant could not interact with RFWD3 (Figure 2E; Figure S2E, Supporting Information), nor could it ubiquitinate RFWD3 as if it had no E3 ligase activity like *UHRF1^CH2A^* (Figure 2F). As expected, its expression led to stabilization of RFWD3 and destabilization of RAD51 (Figure S2F, Supporting Information).

Having established that UHRF1 is RFWD3's E3 in the cell‐based assays, we wanted to determine if UHRF1 can ubiquitinate RFWD3 in vitro in a reconstituted ubiquitination reaction with purified components as we had done previously on DNMT1. ^[^ ^28^ ^]^ To that end, we expressed and purified relevant proteins from *E. coli* (Figure S2G, Supporting Information), and reconstituted the ubiquitination reactions. As shown in Figure S2H (Supporting Information), both UHRF1 and UHRF1^5A^ could ubiquitinate RFWD3 (catalytically inactive RFWD3^C315A^ was used as the substrate to avoid itself‐catalyzed ubiquitination), but RFWD3 interaction‐defective UHRF1^NR2A^ could not. Further, the UHRF1‐catalyzed ubiquitination could be blocked by RAD51 but not by UHRF1 interaction‐defective RAD51^R254Q^ (Figure S2I, Supporting Information). Taken together, these results show that UHRF1 can act as an E3 of RFWD3, both in vivo and in vitro.

Since RFWD3 itself is an E3 ligase, we wondered if it also can ubiquitinate and suppress UHRF1 expression in return. As shown above, the depletion of *RFWD3* did not increase the levels of UHRF1 (Figure 1E). Then we overexpressed *RFWD3*, but no effects on UHRF1 levels were detected either (Figure S2J, Supporting Information), unlike the overexpression of *UHRF1* which brought down RFWD3 levels (Figure S2K, Supporting Information). Taken together, these data indicate that UHRF1 regulates RFWD3 in one direction.

### 2.3 RAD51 Modulates UHRF1 in the Regulation of RFWD3

**Figure 3:**
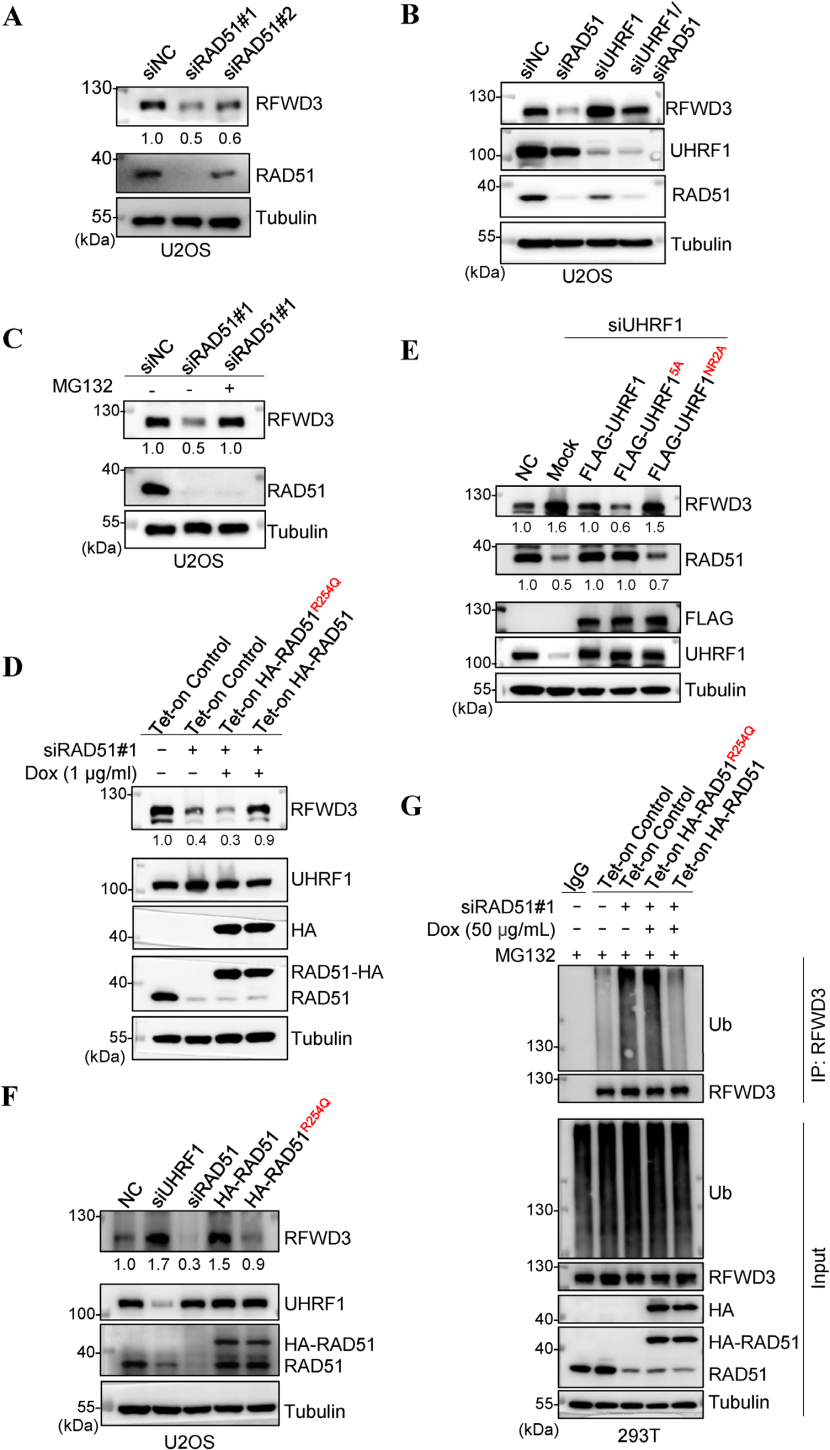
RAD51 modulates UHRF1 in the ubiquitination of RFWD3. A) Western blotting analysis of RFWD3 protein levels in *RAD51*‐depleted U2OS cells. The cells were transfected with scrambled or RAD51 siRNA (two independent siRNAs) for 48 h. B) Western blotting analysis of RFWD3 protein levels after knocking down *UHRF1* and *RAD51*, individually or simultaneously in U2OS cells. C) Western blotting analysis of RFWD3 protein levels in *RAD51*‐depleted U2OS cells. The cells were transfected with scrambled or RAD51 siRNA for 42 h, followed by 6‐hour treatment of 10 µm MG132 or DMSO. D) Western blotting analysis of RFWD3 and UHRF1 protein levels in U2OS cells depleted of *RAD51* and re‐expressing either wildtype *RAD51* or *RAD51R^254Q^*. The cells with inducible expression of HA‐tagged *RAD51* or *RAD51R^254Q^* were transfected with siRAD51 for 24 h and induced to express RAD51 with 1 µg mL^−1^ doxycycline for another 24 h before the analysis. E) Western blotting analysis of RFWD3 and RAD51 protein levels in *UHRF1*‐depleted U2OS cells re‐expressing *UHRF1*, *UHRF1^5A^* or *UHRF1^NR2A^*. F) Western blotting analysis of RFWD3 protein levels in U2OS cells depleted of *RAD51* or *UHRF1*, or overexpressing of *RAD51* or *RAD51^R254Q^*. G) Ubiquitination assay of RFWD3 in *RAD51*‐depleted HEK293T cells re‐expressing *RAD51* or *RAD51^R254Q^*. The cells with inducible expression of HA‐tagged *RAD51* or *RAD51^R254Q^* were transfected with siRAD51 for 24 h and induced to express *RAD51* with 1 µg mL^−1^ doxycycline for another 24 h before the analysis.

We reported before that RAD51 protects another substrate of UHRF1, DNMT1, from ubiquitination and degradation.^[^ ^28^ ^]^ We wondered if RAD51 would do the same for its own destructor RFWD3. Indeed, the depletion of *RAD51* caused ≈50% downregulation of RFWD3 (**Figure** **3**A) and destabilized the E3 ligase (Figure S3A, Supporting Information), which could be rescued by co‐depletion of *UHRF1* (Figure 3B) or by blocking the proteasome (Figure 3C; S3B, Supporting Information). Further, when *RAD51* was knocked down, the re‐expression of wildtype *RAD51* could rescue the levels of RFWD3, but that of *RAD51^R254Q^*, a mutant that cannot interact with UHRF1,^[^ ^28^ ^]^ could not do the same (Figure 3D). Moreover, the re‐expression in *UHRF1*‐depleted cells of *UHRF1^5A^* downregulated (Figure 3E) and destabilized RFWD3 (Figure S3C, Supporting Information). Here again, the RFWD3 interaction‐deficient *UHRF1^NR2A^* upregulated RFWD3 and downregulated RAD51 as expected (Figure 3E). On the other hand, overexpressing *RAD51* could upregulate RFWD3 as expected, but overexpressing *RAD51^R254Q^* could not (Figure 3F). In this experiment, we also depleted *UHRF1* and *RAD51* for comparison. Assuringly, the former upregulated and the latter downregulated RFWD3 (Figure 3F). These data suggest that RAD51 inhibits UHRF1‐mediated ubiquitination and degradation of RFWD3, much like it does for DNMT1.^[^ ^28^ ^]^ We therefore examined the ubiquitination levels on RFWD3 in the cells with *RAD51* depletion and with or without re‐expression of the recombinase. As shown in Figure 3G, the depletion of *RAD51* resulted in increased ubiquitination of RFWD3, which was suppressed by the re‐expression of wildtype *RAD51* but not by that of *RAD51^R254Q^*, indicating that RAD51 has to interact with UHRF1 to exsert its protection for RFWD3.

**Figure 4:**
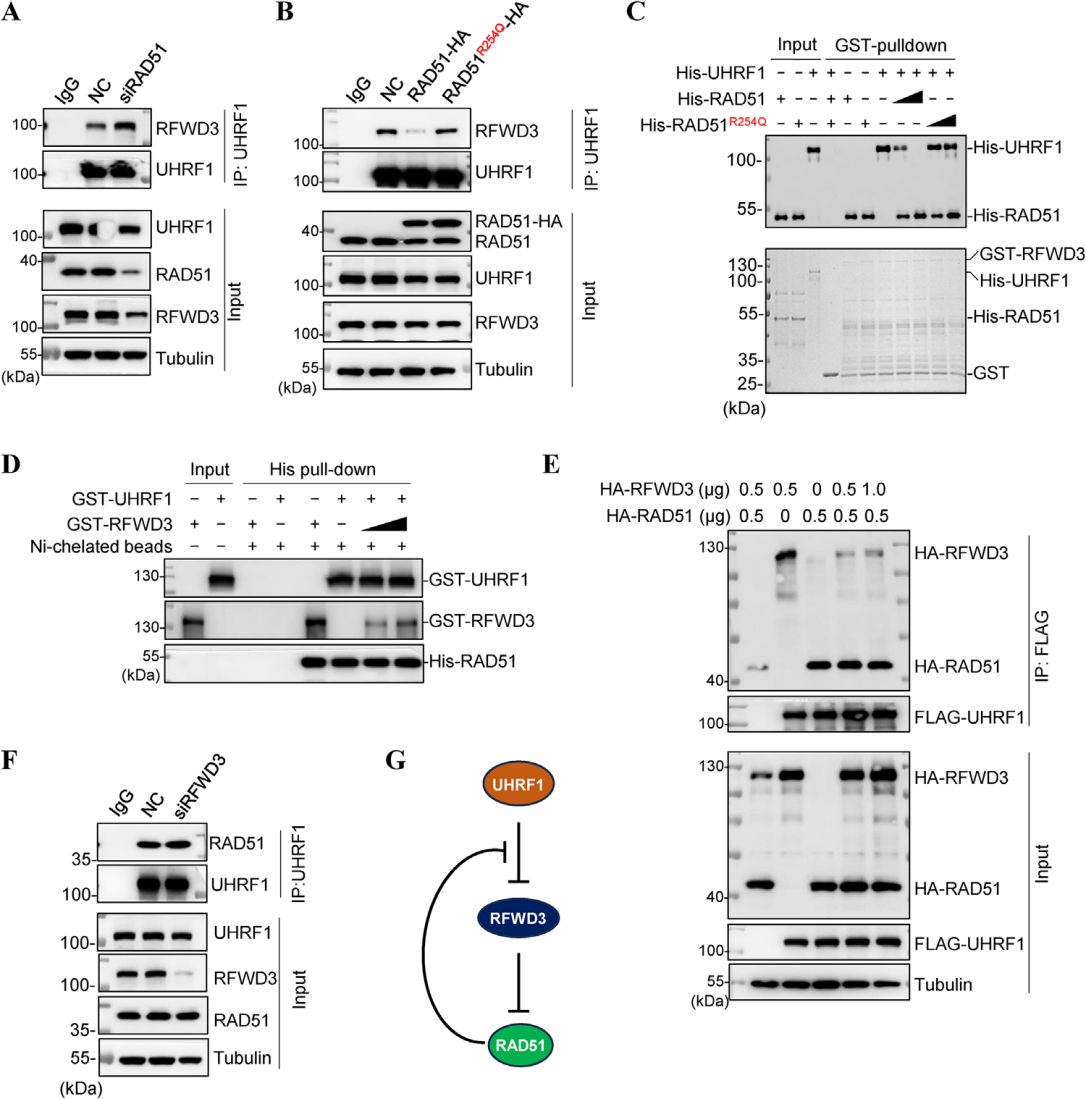
RAD51 inhibits UHRF1 from binding to RFWD3. A) Immunoprecipitation analysis of the interaction between endogenous UHRF1 and RFWD3 in HEK293T cells depleted of *RAD51* expression. B) Immunoprecipitation analysis of the interaction between endogenous UHRF1 and RFWD3 in HEK293T cells overexpressing *RAD51* or *RAD51^R254Q^*. C) GST pull down assay using GST‐RFWD3 or GST to precipitate His‐UHRF1, His‐RAD51, or His‐RAD51^R254Q^. His‐tagged proteins were detected with western blotting (the upper panels). The bottom panel shows Coomassie blue staining of the purified proteins used in the assay. D) His‐pull down assay using His‐RAD51 to precipitate GST‐RFWD3 or GST‐UHRF1. Ni‐chelated beads were used as a negative control. The pull‐down products were detected by Western blotting. E) Immunoprecipitation analysis of the interaction between ectopically expressed RFWD3‐HA or RAD51‐HA with FLAG‐UHRF1 in HEK293T cells. F) Immunoprecipitation analysis of the interaction between endogenous UHRF1 and RAD51 in HEK293T cells depleted of *RFWD3* expression. G. Schematic drawing of the UHRF1‐RFWD3‐RAD51 triple negative feedback loop.

We next asked how RAD51 exserts this protection. Given that the site that interacts with RAD51 in UHRF1 resides between residues 745–750,^[^ ^28^ ^]^ very close to the site that interacts with RFWD3 (between 791 and 793, Figure 2D), we postulated that binding RAD51 prevents UHRF1 from binding RFWD3. Indeed, the depletion of *RAD51* enhanced the interaction between UHRF1 and RFWD3 (**Figure** **4**A), while the overexpression diminished the interaction (Figure 4B). As expected, the overexpression of UHRF1 interaction‐deficient *RAD51^R254Q^* could not do that (Figure 4B). The i*n vitro* GST pulldown assay also demonstrated that RAD51 could drive away RFWD3 from interacting with UHRF1, but RAD51^R254Q^ could not (Figure 4C). Further, using purified RAD51 (His‐RAD51) to pulldown RFWD3 and UHRF1, we saw a strong interaction between RAD51 and UHRF1 that was not affected by increasing amounts of RFWD3 (Figure 4D). Similar results were obtained with transfections in 297T cells (Figure 4E). On the other hand, depleting *RFWD3* did not alter the interaction between UHRF1 and RAD51 (Figure 4F), indicating that RFWD3 does not affect the binding of RAD51 by UHRF1. These data indicate that UHRF1 binds RAD51 much stronger than RFWD3, and RAD51 can drive away RFWD3 from UHRF1 but RFWD3 cannot do the same to RAD51.

Taken together, these results demonstrate a triple negative feedback circuit amongst UHRF1, RFWD3 and RAD51 which ensures appropriate levels of RAD51 and RFWD3 are maintained in cells (Figure 4G).

### 2.4 The Ubiquitination of RFWD3 is Regulated by the Phosphorylation Status of UHRF1

It was reported previously that UHRF1 is phosphorylated by cyclin‐dependent kinases (CDKs) in S and M phases of cell cycle. Two CDK consensus phosphorylation sites (Ser639 and Ser661, on isoform 1: NP_0 012 76981.1) were reported to be phosphorylated in S phase (S661) ^[^ ^22^ ^]^ and M phase (S639).^[^ ^32^ ^]^ S661 phosphorylation is required for the interaction of UHRF1 with BRCA1 and is involved in repair pathway choice,^[^ ^22^ ^]^ while S639 phosphorylation mediates the interaction with USP7.^[^ ^32^ ^]^ The phosphorylation of zebra fish UHRF1 at the equivalent S661 site is essential for development.^[^ ^33^ ^]^ We therefore wanted to know if S661 phosphorylation impacts the UHRF1‐RFWD3‐RAD51 axis. We expressed *UHRF1, UHRF1^S661A^* and *UHRF1^S661D^*, and examined the interactions amongst the three. As shown in **Figure** **5**A, the un‐phosphorylatable UHRF1 mutant protein (S661A) interacted less with RFWD3 but more with RAD51 when compared to the wildtype protein. On the other hand, the phosphorylation‐mimicking mutant (S661D) behaved just the opposite, interacting more with RFWD3 and less with RAD51 (Figure 5A). Thus, Ser661 phosphorylation does affect how UHRF1 interacts with its prey RFWD3 as well as with its inhibitor RAD51. This result predicts that the more phosphorylation of UHRF1 the more RFWD3 ubiquitination and degradation. Indeed, *UHRF1^S661D^* promotes the ubiquitination of RFWD3 while *UHRF1^S661A^* is unable to (Figure 5B). As a result, we could see a reduction in RFWD3 levels in *UHRF1^S661D^*‐expressing cells (Figure 5C).

Figure 5RFWD3 ubiquitination is regulated by the phosphorylation status of UHRF1. A) Immunoprecipitation analysis of the interaction between ectopically expressed RFWD3‐HA or RAD51‐HA with FLAG‐UHRF1, FLAG‐UHRF1^S661A^ or FLAG‐UHRF1^S661D^ in HEK293T cells. B) Ubiquitination assay of RFWD3 in *UHRF1*‐depleted HEK293T cells re‐expressing *UHRF1, UHRF1^S661A^* or *UHRF1^S661D^*. C) Western blotting analysis of RFWD3 protein levels in *UHRF1*‐depleted U2OS cells re‐expressing *UHRF1*, *UHRF1^S661A^* or *UHRF1^S661D^*. D) Western blotting analysis of RFWD3 protein levels in U2OS cells depleted of *UHRF1*, *PP4C* or *PP4R3A*. E) Immunoprecipitation analysis of the interaction between endogenous UHRF1 and RFWD3 in HEK293T cells depleted of *PP4C* expression. F) Immunoprecipitation analysis of the interaction between ectopically expressed UHRF1‐HA and FLAG‐PP4R3A in HEK293T cells. G) Analysis of UHRF1 phosphorylation. HEK293T cells were transfected with plasmids for *FLAG‐UHRF1*, *FLAG‐UHRF1^FP2A^* or *FLAG‐UHRF1^F757/AP760A^* expression. The FLAG‐tagged proteins were immunoprecipitated and blotted with phospho‐Ser/Thr antibodies. H) Immunoprecipitation analysis of the interaction between ectopically expressed FLAG‐PP4R3A and UHRF1‐HA or UHRF1^FP2A^‐HA in HEK293T cells. I) Western blotting analysis of RFWD3 protein levels in *UHRF1*‐depleted U2OS cells re‐expressing *UHRF1* or *UHRF1^FP2A^*. J) A schematic representation of the UHRF1 protein depicting its functional domains and binding interfaces, including residues involved in interactions with RAD51, RFWD3, BRCA1, USP7, PP4R3A.

**Figure:**
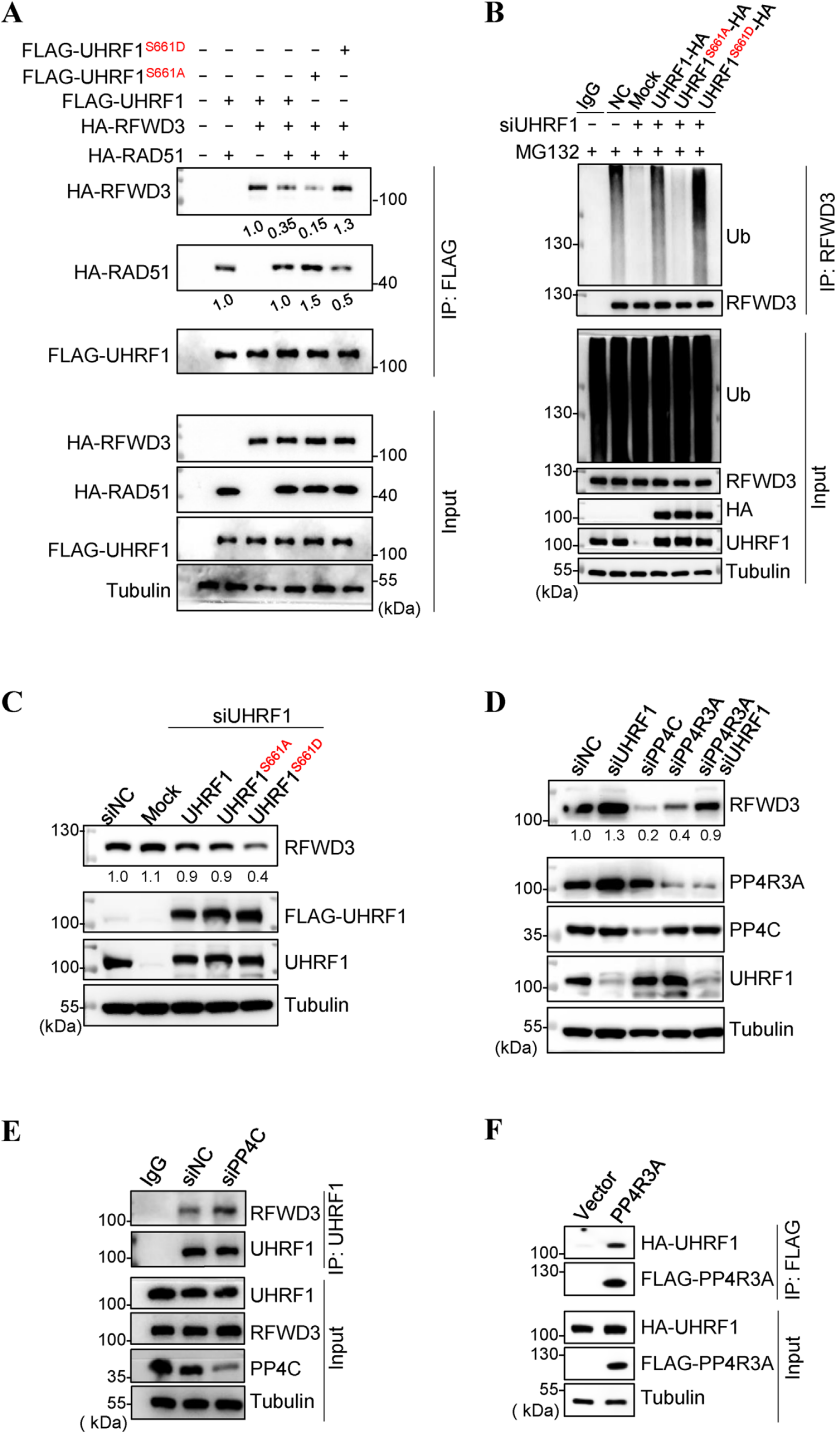

**Figure:**
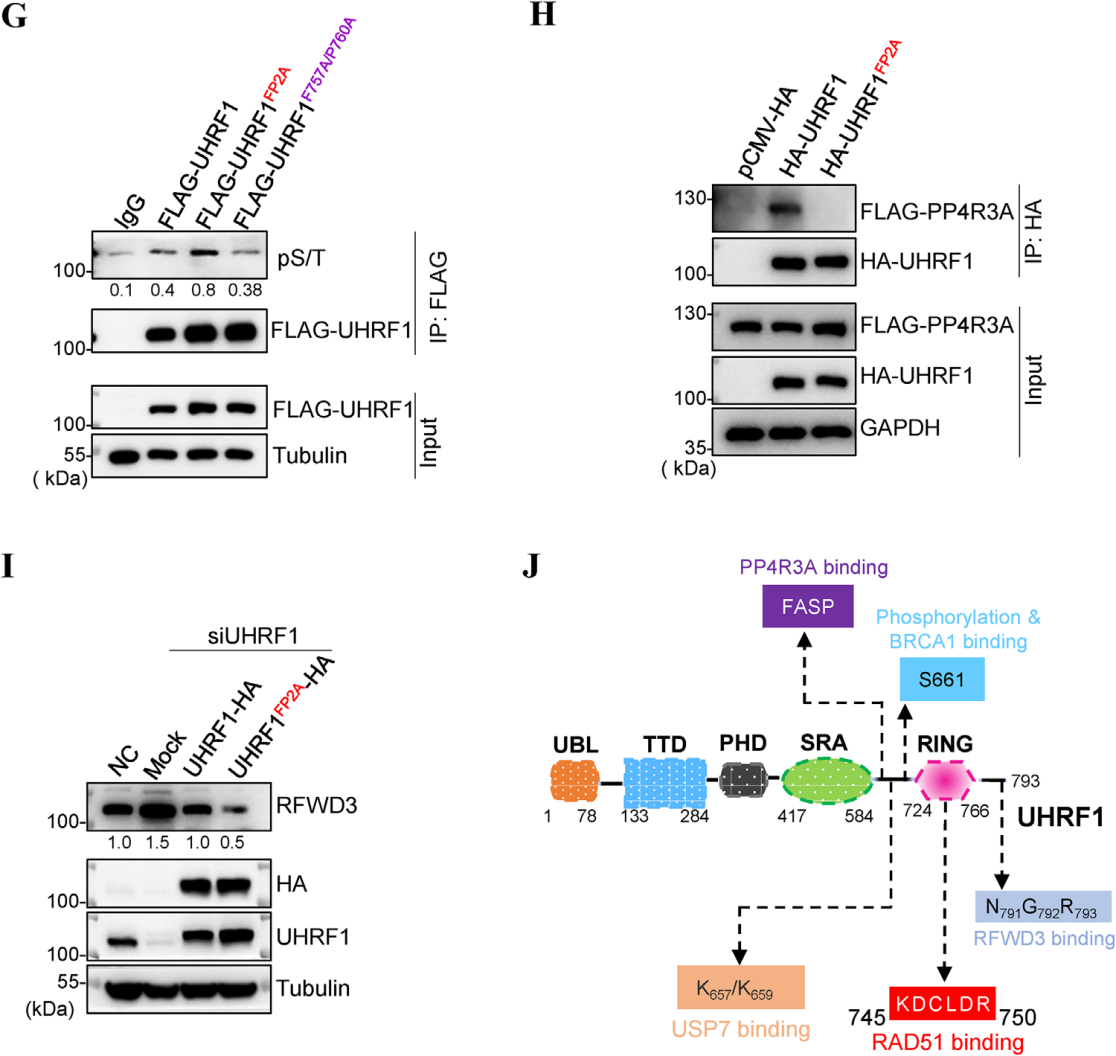

The phosphorylation of UHRF1 may also allosterically affect its E3 activity toward RFWD3, in addition to affecting the interaction between the two. To find out if that is the case, we expressed and purified UHRF1 (WT, S661A, and S661D) (Figure S4A, Supporting Information) and performed in vitro ubiquitination assays (Figure S4B,C, Supporting Information). It is clear that the status at S661 has little effect on UHRF1's E3 activities, either toward itself (Figure S4B, Supporting Information) or toward RFWD3 (Figure S4C, Supporting Information). The E3‐dead RFWD3 mutant (C315A) was used as the substrate to avoid RFWD3 self‐ubiquitination.

To further demonstrate that phosphorylation regulates UHRF1, we treated U2OS cells with a selective CDK2 inhibitor (CDK2i), CDK2‐IN‐4,^[^ ^34^ ^]^ to block UHRF1 phosphorylation and then examined the effects on RFWD3 expression. As shown in Figure S5A (Supporting Information), CDK2i could upregulate the protein levels of RFWD3 in a *UHRF1*‐dependent manner. The inhibitor also extended the half‐life of RFWD3 (Figure S5B, Supporting Information). In addition, we constructed a fusion between cyclin D1 and CDK2 (CCND1‐CDK2) to make CDK2 constitutively active as reported.^[^ ^35^ ^]^ The expression of this fusion protein resulted in downregulation of RFWD3 and could overcome the CDK2i’ s effect on RFWD3 (Figure S5C, Supporting Information), suggesting that the CDK2i we used acts through CDK2, not through other CDKs like CDK1. Taken together, these data strongly support the notion that phosphorylation of UHRF1 at S661 by CDK2 enhances its interaction with RFWD3 and the ubiquitination‐mediated degradation of the latter.

Next, we wanted to determine which phosphatase is responsible for dephosphorylating UHRF1. Previous work by others suggested CDC14A, CDC14B, and protein phosphorylase 4 (PP4) as phosphatases to undo CDK phosphorylation. We therefore knocked them down and examined the expression levels of RFWD3. It turned out that only the depletion of *PP4C* (the catalytic subunit of PP4) expression resulted in the downregulation of RFWD3 (Figure S5D, Supporting Information). Such downregulation could be mimicked by depleting the regulatory subunit of PP4, PP4R3A, and could be rescued by simultaneous depletion of *UHRF1* and *PP4R3A* (Figure 5D). As expected, when *PP4C* was knocked down, while the phosphorylation of UHRF1 increased (Figure S5E, Supporting Information), the interaction between UHRF1 and RFWD3 also increased (Figure 5E). Further, we could detect an interaction between UHRF1 and PP4R3A (Figure 5F), whereas no interaction between PP4R3A and RFWD3 could be detected (Figure S5F, Supporting Information).

Having established PP4 as a phosphatase of UHRF1, we wanted to determine the site in UHRF1 that interacts with PP4. It was reported that PP4 (through its regulatory subunit R3A) binds to FxxP motif of its substrates.^[^ ^36^ ^]^ UHRF1 contains 2 such motifs, FASP (residues 637–640), and FSCP (residues 757–760). We mutated these two motifs (FASP to AASA, and FSCP to ASCA) and examined their phosphorylation. Only the first motif (FASP) seemed important for the phosphorylation (Figure 5G). Mutating it also abolished the interaction between UHRF1 and PP4R3A (Figure 5H), but enhanced the interaction between UHRF1 and RFWD3 as the mutant could not be dephosphorylated anymore (Figure S5G, Supporting Information). Consistent with these observations, this mutant (UHRF1^FP2A^) was capable of downregulating RFWD3 (Figure 5I). We further treated U2OS cells with a potent PP4 inhibitor, fostriecin.^[^ ^37^ ^]^ The treatment destabilized RFWD3, contrasting the effect of CDK2i (Figure S5H, Supporting Information). Inhibition of PP4 also increased the phosphorylation of UHRF1, again opposite to CDK2i treatment (Figure S5I, Supporting Information).

The various interacting patterners of and the interacting sites on UHRF1 as reported and identified here are shown in Figure 5J.

### 2.5 UHRF1 Protects RAD51 at DNA Damage Sites

It is known that RFWD3 removes RAD51 from DNA by the end of homologous recombination process.^[^ ^27^ ^]^ What prevents it to do so before RAD51 completes strand searching and pairing is unknown. With the results we obtained so far, it is reasonable to hypothesize that it is UHRF1 that holds RFWD3 back. To demonstrate that, we examined RFWD3 kinetics at damage sites in cells treated with mitomycin (MMC) for 1 hour and then released. MMC treatment resulted in numerous DNA damages sites which could be visualized through immunofluorescent staining of γH2AX (Figure S6A, Supporting Information). The staining diminished as the cells were released from MMC treatment (Figure S6A, Supporting Information). As expected, RFWD3 co‐stained with γH2AX over the course of MMC treatment and release (Figure S6A, Supporting Information). These cells were depleted of *UHRF1* but supplemented with exogenous *UHRF1*, *UHRF1^5A^* or *UHRF1^NR2A^* (Figure S6B, Supporting Information). Right after the MMC treatment, RFWD3 foci could be readily detected in control cells, but hardly detectable in *UHFR1‐*depleted cells (**Figure** **6**A). The same is true for RAD51 (Figure 6B). This is not unexpected given that UHRF1 recognizes interstrand crosslink (ICL) lesions and activates Fanconi pathway ^[^ ^19^, ^20^ ^]^ and that RPA (replication protein A) recruits RFWD3 to DNA damage sites.^[^ ^38^ ^]^ While re‐expressing wildtype *UHRF1* restored RFWD3 foci as expected, re‐expressing *UHRF1^5A^* could not (Figure 6A), most likely because this mutant causes excess RFWD3 degradation (Figure 3E). This caused RAD51 foci to persist in *UHRF1^5A^*‐expressing cells (Figure 6B), confirming the previous report that RAD51 is removed by RFWD3. ^[^ ^27^ ^]^ Of note, *UHRF1^5A^* did not increase appreciably the total levels of RAD51 (Figures 1, 3; Figure S6B, Supporting Information). Moreover, in the cells re‐expressing *UHRF1^NR2A^*, a mutant that cannot interact with RFWD3 (Figure 2E), RFWD3 foci not only were restored but also stayed much longer than those in the other cells (Figure 6A), which strongly suggests that UHRF1 functions to restrain RFWD3 at the damage sites. In these cells, RAD51 foci were hardly detectable even at the time of releasing (Figure 6B), which is likely a result of reduced RAD51 levels in these cells to begin with (Figure 3E).

Figure 6UHRF1 restrains RFWD3 to protect RAD51 at DNA damage sites. A) Representative Immunofluorescence (IF) images of RFWD3 foci. U2OS cells depleted of *RFWD3* or *UHRF1* and re‐expressing FLAG‐tagged *UHRF1*, *UHRF1^5A^*, or *UHRF1^NR2A^* were treated with 1 µm MMC for 1 h and released into fresh culture media. The cells were harvested at the indicated time of release and processed for immunofluorescent staining for RFWD3. The number of RFWD3 foci/cell were counted and quantified from 100 FLAG IF‐positive cells. B) Representative images of RAD51 foci in the cells of (A). The number of RAD51 foci/cell were counted and quantified from 100 FLAG IF‐positive cells. C) Representative of RFWD3 foci. U2OS cells were treated with 1 µm MMC for 1 h and released into fresh culture media containing 4 µm CDK2i. The cells were harvested at the indicated time of release and processed for immunofluorescent staining for RFWD3. The number of RFWD3 foci/cell were counted and quantified from 100 cells. D) Representative of RFWD3 foci. U2OS cells were treated with 1 µM MMC for 1 h and released into fresh culture media containing 6 µM PP4i. The cells were harvested at the indicated time of release and processed for immunofluorescent staining for RFWD3. The number of RFWD3 foci/cell was counted and quantified from 100 cells. Data (mean ± S.D.) from 3 independent experiments are presented. E.)Relative homologous recombination (HR) efficiency. The HR‐reporter cells (U2OS containing DR‐GFP) were depleted of *UHRF1* expression and made re‐expressing *UHRF1, UHRF1^5A^, or UHRF1^NR2A^*. Data represent the mean ± SD from three independent experiments normalized to the control cells (siNC). * Indicates *p* <0.05; ** *p* <0.01;*** *p* <0.001 and **** *p* <0.0001. Scale bar, 5 µm.

**Figure:**
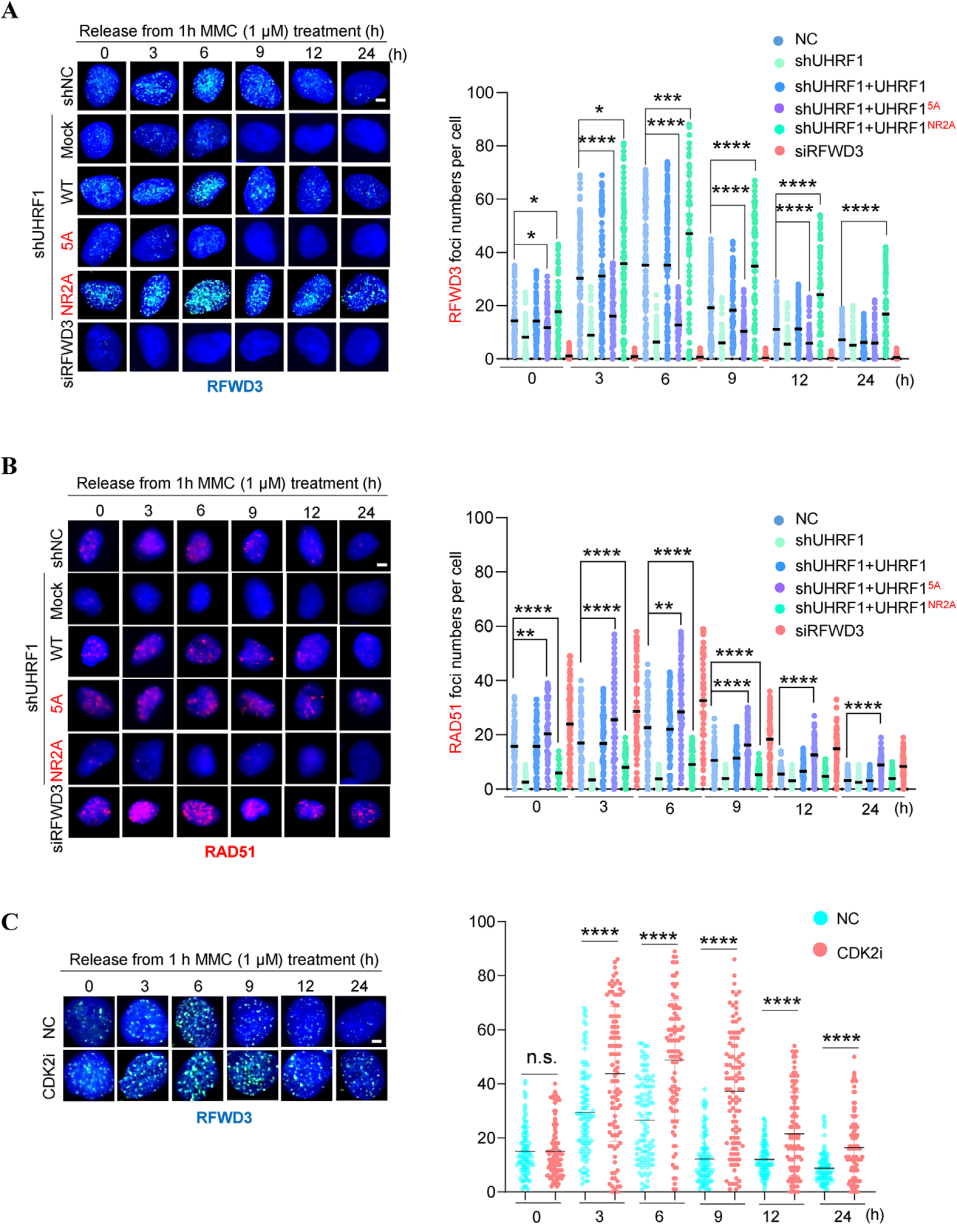

**Figure:**
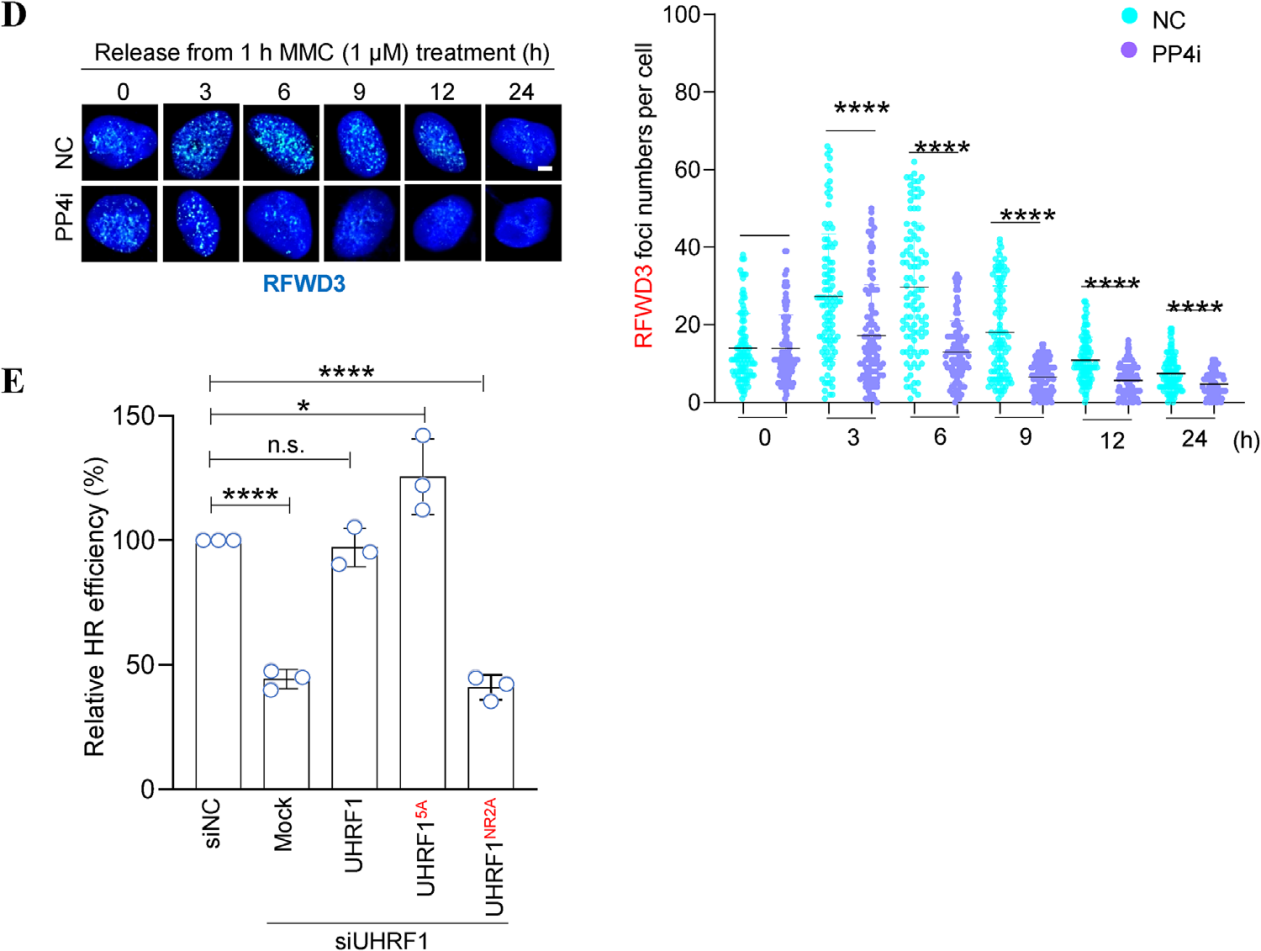

Since CDK2‐mediated phosphorylation of UHRF1 regulates its interaction with RFWD3, we wondered if such phosphorylation plays a role in protecting RAD51 from RFWD3. To that end, we added CDK2i to the cells right at the beginning of releasing from 1 hour MMC treatment, and examined the kinetics of RFWD3 and RAD51 foci. As shown in Figure 6C, it is clear that inhibiting CDK2 resulted in very similar RFWD3 kinetics as the expression of *UHRF1^NR2A^* (Figure 6A). More importantly, under these conditions, RAD51 foci were the same (and they should) at the beginning of releasing between control and CDK2i‐treated cells, but over time it became evident that RAD51 foci disappeared much faster in CDK2i‐treated cells than in control cells (Figure S6C, Supporting Information). In contrast, adding PP4i at the beginning of the release from MMC treatment caused premature disappearance of RFWD3 foci (Figure 6D) and slower clearance of RAD51 (Figure S6D, Supporting Information). These results strongly argue that it is the phosphorylated UHRF1 that protects RAD51 by restraining RFWD3 at damage sites. Blocking UHRF1 phosphorylation leads to higher levels of RFWD3 and premature degradation of RAD51 ensued, whereas enhancing UHRF1 phosphorylation (via inhibiting PP4) results in excess degradation of RFWD3 and delayed removal of RAD51.

Using the same strategy (depleting *UHRF1* and re‐expressing *UHRF1* wildtype or mutants), we examined the kinetics of RFWD3 at DNA damage sites in U2OS cells treated with hydroxy urea (HU, 2 mM) for 24 h and released (Figure S6E–G, Supporting Information). Very similar results were obtained as in the cells treated with MMC and released (Figure 6A).

Having established a critical role of UHRF1 in controlling RFWD3, we next wanted to determine whether disrupting such a role would interfere with HR repair. We therefore took advantage of the HR reporter cell line U2OS/GFP‐DR.^[^ ^39^ ^]^ We depleted *UHRF1* and re‐expressed wildtype or mutant *UHRF1* in these cells (Figure S6H, Supporting Information). As shown in Figure 6E, depleting *UHRF1* reduced the HR efficiency by ≈50%, and the re‐expression of wildtype *UHRF1* restored the HR efficiency. Interestingly, the re‐expression of *UHRF1^5A^* increased the HR efficiency by ≈25%, comparing to the cells re‐expressing wildtype *UHRF1*. This is understandable as RAD51 in *UHRF1^5A^*‐expressing cells stayed longer at damage sites (Figure 6B). Not unexpectedly, the re‐expression of *UHRF1^NR2A^* could not restore HR efficiency at all, most likely because RAD51 was removed from damage sites precociously by the increased presence of RFWD3 (Figure 6A,B).

## 3 Discussion

**Figure 7:**
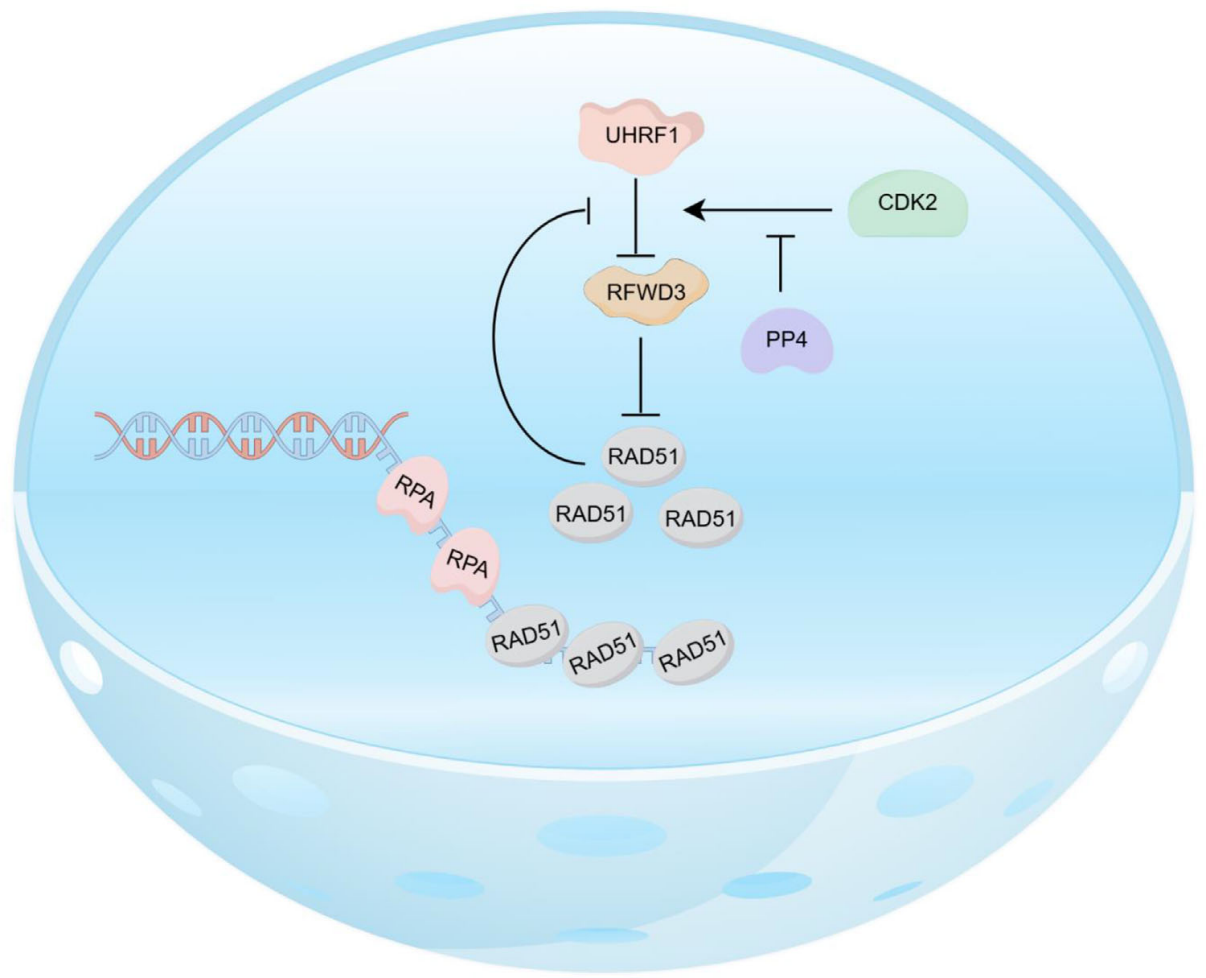
A model illustrating the control of RFWD3 by UHRF1 at DNA damage sites. The phosphorylation of UHRF1 provides a control to cells over when to retrain RFWD3 and when to release it from UHRF1 so that RAD51 can be cleared away for the completion of HR repair.

Homologous recombination‐mediated repair of double strand breaks is essential for genome stability. It is a multi‐step process requiring a large number of proteins to work together in a coordinated manner. In the last few steps of the process, RAD51 is loaded onto single strand DNA to form protein‐DNA filaments for strand invasion and paring with homologous sequences, and is removed afterwards by RFWD3‐mediated ubiquitination.^[^ ^27^ ^]^ However, it is unclear what prevents RFWD3 to do so before homology searching and pairing. Here we provide strong evidence to support the notion that it is UHRF1, an E3 ubiquitin ligase itself, that keeps RFWD3 in check (**Figure** **7**). Without such a check, RAD51 is removed precociously (Figure 6B). This check we believe is dynamically regulated by phosphorylation on UHRF1 through the actions of CDK2 and PP4 (Figure 7). On top of that, both CDK2 and PP4 might be regulated to achieve fine tuning of UHRF1 activity.

Since the first report in 2002 that *Uhrf1*‐deficient murine embryonic stem cells were sensitive to DNA damage agents and replication blocks,^[^ ^40^ ^]^ UHRF1's roles in DNA damage and replication stress responses have been extensively studied. These studies revealed a number of UHRF1‐interacting partners functioning in DNA damage response pathways including BRCA1. We showed recently that UHRF1 interacts directly with yet another DNA response core protein RAD51 and demonstrated the importance of this interaction in the maintenance methylation of DNA.^[^ ^28^ ^]^ We show here that this interaction is also implicated in DNA damage repair as the interaction helps maintain the protein level of RFWD3, another E3 ligase heavily involved in DNA damage and replication stress responses. Ironically, RAD51, a substrate of RFWD3, protects its own destructor from being destructed by UHRF1, resulting in a triple‐negative feedback circuit (Figure 4G). We provide ample evidence that this regulatory circuit is critical in maintaining the levels of RAD51 and RFWD3 in unperturbed cells as well as in damaged cells. At DNA damage sites, this regulatory circuit is likely to be complicated by several factors. First, UHRF1 is very likely bound by histone H3, and we know H3‐binding excludes RAD51 from interacting with UHRF1.^[^ ^28^ ^]^ Second, in the case of interstrand crosslink, UHRF1 directly binds to the lesion,^[^ ^19^, ^20^ ^]^ which might affect UHRF1's ability to interact with RAD51 or to ubiquitinate RFWD3. Despite all these, however, there must be free UHRF1 molecules around that need to be inhibited by RAD51, otherwise we would not have seen the effect of UHRF1^5A^ on RFWD3 (Figure 6A). In addition, RFWD3 is recruited to DNA damage sites through interacting with RPA.^[^ ^38^ ^]^ It remains to be determined if the interaction with RPA affects UHRF1 regulation of RFWD3 or RFWD3 regulation of RAD51.

It has been increasingly recognized that cyclin‐dependent kinases, especially CDK2, play important roles in DNA damage response (DDR).^[^ ^41^, ^42^, ^43^ ^]^ It was reported that DNA damage repair was impaired in *Cdk2* ^‐/‐^ cells, and as a result these cells sustain more DNA damage and *Cdk2‐*deficient mice were more sensitive to lethal irradiation than the wildtype control animals.^[^ ^44^, ^45^ ^]^ A number of proteins involved in DDR were found phosphorylated by CDK2 including BRCA1/2,^[^ ^45^, ^46^ ^]^ ATRIP ^[^ ^47^ ^]^ and UHRF1,^[^ ^22^ ^]^ just to name a few. Here we provide yet another piece of evidence supporting the importance of CDK2 in DDR. We believe CDK2 keeps RFWD3 away from RPA and RAD51 early on during the repair process by activating RFWD3 ubiquitin ligase UHRF1, especially considering that RAD51 is likely no longer bound to and inhibiting UHRF1 anymore (see above). We believe that once RAD51‐coated single strand DNA completes strand invasion and pairing, CDK2 is inactivated or PP4 is activated via unidentified mechanism(s), resulting in dephosphorylation and hence inactivation of UHRF1. As a result, RFWD3 is now allowed to remove RAD51 away to complete homologous recombination repair process. Of note, in the experiments (Figure 6) we were not looking at each individual focus (RFWD3's or RAD51's) from beginning to the end, which is technically impossible at the moment. Instead, we looked at the whole population of foci and relied on the power of statistics to draw conclusions. In addition, the PP4 inhibitor fostriecin we used also inhibits PP2A potently.^[^ ^48^ ^]^ Thus, the observed effect on RFWD3 dynamics (Figure 6D) might come partly from inhibiting PP2A.

The E3 ligase activity of UHRF1 must be tightly controlled both in time and in space as it works in a number of different processes. A novel regulating mechanism we discovered is the inhibition by RAD51.^[^ ^28^ ^]^ This mechanism applies also to RFWD3 as we unveiled here. UHRF1‐RFWD3‐RAD51 in fact constitutes a feedback circuit (Figure 4G) that ensures appropriate levels of RFWD3 and RAD51 in the cell. This feedback loop likely operates locally at damage sites as well, and together with the phosphorylation of UHRF1, it ensures proper levels of RAD51 for HR and the timing of RAD51 removal (Figure 7).

## 4 Experimental Section

### Cell Culture and Reagents

HEK293T (RRID: CRL‐3216) and U2OS cells (RRID: HTB‐96) were purchased from ATCC and cultured in Dulbecco's modified Eagle's medium (DMEM) containing 10% fetal bovine serum (FBS) in the presence of 1% penicillin/streptomycin (P/S) at 37 °C with 5% CO2. The U2OS DR‐GFP cell line was kindly provided by Dr. Jun Huang (Zhejiang University).

siRNA transfection was performed with Lipofectamine RNAiMAX (Invitrogen) according to the manufacture’ protocol.

Antibodies, chemicals and the sequences of PCR primers, siRNA, and shRNA used in this study are listed in the supplementary reagent table.

### RNA Isolation and Quantitative RT‐PCR

RNA was isolated with the TRIzol (Sigma‐Aldrich, America) method following manufacturer's instructions. cDNA was generated using a reverse transcription kit (Vazyme, Nanjing, China) and real‐time PCR was performed with ChamQ universal SYBR GreenER qPCR Master Mix (Vazyme, Nanjing, China) following manufacturer's instructions.

### Immunoprecipitation, GST Pulldown, and Western Blotting Analysis

For immunoprecipitation, the cells were harvested and washed twice with cold PBS and then lysed with NETN buffer (20 mm Tris‐HCl (pH 7.5), 1 mm EDTA, 100 mm NaCl and 0.5% NP‐40) containing protease and phosphatase inhibitors cocktails on a rotator at 4 °C for 30 min. The cell lysates were cleared by centrifugation and incubated with appropriate antibodies at 4 °C overnight with agitation. Prrotein A/G agarose beads were then added and further incubated for another 4 h at 4 °C on a rotator. Subsequently, the beads were washed with NETN buffer containing 200 mm NaCl for three times and analyzed by western blotting. For tagged proteins, we used anti‐FLAG affinity beads (GenScript) or anti‐HA affinity beads (Sigma) with for 3 h incubation at 4 °C on a rotator. The bead‐bound proteins were washed with NETN buffer containing 200 mm NaCl for three times and then boiled in 2 × SDS buffer for 10 min. The bound proteins were subjected to western blotting analysis.

For western blot analysis, the cells were lysed in RIPA buffer with protease inhibitors, and the lysates were centrifuged to remove debris. Protein concentration was determined with BCA assay. Equal protein amounts were boiled in SDS loading buffer, separated on an SDS‐PAGE gel, and transferred to nitrocellulose membranes. The membranes were blocked with 5% milk in TBST, incubated with primary antibodies overnight at 4 °C, extensively washed, and then incubated with HRP‐conjugated secondary antibodies for 1 h. After washing, the membranes were visualized using a chemiluminescent substrate.

### Immunofluorescent Staining

The cells were cultured on coverslips for 24 h and then washed with cold PBS twice, fixed with PIEMF buffer (20 mm PIPES pH 6.8/10 mm EGTA/0.5%/TritonX‐100/1mM MgCl_2_/4% formaldehyde) for 10 min at room temperature. After fixation, the cells were permeabilized in a 0.5% Triton X‐100% solution for 10 min and then blocked with 3% BSA for 1 h at room temperature. The coverslips were then incubated with primary antibodies overnight at 4 °C, washed with 3% BSA in PBST buffer three times, incubated with fluorescent dye‐conjugated secondary antibodies for 40 min and washed. The coverslips were mounted onto glass slides using DAPI‐containing anti‐fade solution and visualized under an Olympus BX53 fluorescence microscope.

### Recombinant Protein Preparation and Purification

The recombinant proteins, including GST‐RFWD3, GST‐RFWD3^C315A^, GST‐UHRF1, GST‐RAD51, His‐RAD51, His‐RAD51^R254Q^, His‐UHRF1, His‐UHRF1^NR2A^, His‐UHRF1^S661A^, His‐UHRF1^S661D^ and various His‐UHRF1 truncations (1–724, 1–745, 1–750, 1–753, and 1–755), were expressed in *Escherichia coli* BL21(DE3) cells with 0.2 or 0.5 mm isopropyl β‐D‐1‐thiogalactopyranoside (IPTG) for 18 h at 16 °C for induction. The *E. coli* cells were harvested by centrifugation at 7000 g for 5 min at 4 °C. The cell pellets were washed five times with cold phosphate‐buffered saline (PBS) and resuspended in a lysis buffer composed of 50 mm Tris‐HCl (pH 7.4), 100 mm NaCl, 1 mM EDTA, 10% glycerol. A protease inhibitor cocktail was added to prevent protein degradation. The cell suspension was sonicated and centrifuged at 16 000 g for 15 min at 4 °C.

For the purification of GST‐tagged proteins, the *E. coli* cell lysates prepared above were incubated with Glutathione resin (GenScript) equilibrated with five bed volumes of cold PBS for 1 h on a rotator at 4 °C. The resin beads were washed with 20 bed volumes of cold wash buffer A (50 mm Tris‐HCl, pH 8.0, 500 mm NaCl, 1 mm DTT, 1 mm EDTA, 0.5% Triton X‐100), and then re‐equilibrated with wash buffer B (50 mm Tris‐HCl, pH 8.0, 100 mm NaCl, 1 mm DTT, 1 mm EDTA, 0.1% Triton X‐100). Elution of the GST‐fusion protein was achieved with the elution buffer containing 50 mm Tris‐HCl pH8.0/20 mm reduced glutathione plus a protease inhibitor cocktail. The eluted proteins were concentrated through a 30‐kDa cut‐off ultra‐filter tube (Millipore) and stored at −80 °C before use.

His‐tagged proteins were purified similarly with Ni‐NTA agarose beads (Vazyme, Nanjing, China).

### GST Pull‐Down Assay

The purified GST‐tagged proteins were immobilized on 30 µL of Glutathione resin (GenScript). Following immobilization, the resin was washed with BCA buffer (20 mm Tris‐HCl, pH 7.4, 100 mm NaCl, 1 mm DTT, 0.5 mM EDTA, 10% glycerol, 0.1% Triton X‐100, supplemented with a protease inhibitor cocktail). Subsequently, the resin was resuspended in BCA buffer and incubated with appropriate amounts of purified His‐tagged or other non‐GST‐tagged proteins at 4 °C for 3 h on a rotator. The resin‐bound complexes were then extensively washed with BCB buffer (20 mm Tris‐HCl, pH 7.4, 300 mm NaCl, 1 mm DTT, 0.5 mm EDTA, 10% glycerol, 0.1% Triton X‐100, supplemented with a protease inhibitor cocktail). The proteins bound to the resin were eluted by boiling and subsequently detected using western blotting or Coomassie brilliant blue staining of the SDS‐PAGE gels. In some cases, the resin‐bound proteins were used directly.

### His‐Tagged Protein Pull‐Down Assay

For the Ni‐NTA pull‐down assays, Ni‐chelated beads (Sigma‐Aldrich) were equilibrated three times with Binding Buffer A (50 mm Tris‐HCl pH 7.5–8.0/50 mm NaC/10% glycerol/0.1% Triton X‐100/1 mm dithiothreitol), and incubated with purified his‐tagged proteins for 1 h at 4 °C with continuous rotation. The beads were then subjected to five times of washes with 1 mL of Binding Buffer A supplemented with 20 mm imidazole. Proteins bound to the nickel beads were incubated with elution buffer composed of Buffer A plus 250 mm imidazole for 20 min at 4 °C to elute the bound proteins. The eluted proteins were subsequently mixed with 2× loading buffer and incubated at 95 °C for 5 min, separated with SDS‐PAGE electrophoresis followed with western blotting or Coomassie brilliant blue staining.

### In Vivo Ubiquitination Assay

HEK293T cells transfected with various expressing plasmids were treated with 10 µm MG132 for a duration of 6 h before harvesting. The cells were lysed using NETN buffer (20 mm Tris‐HCl pH 7.5/1 mm EDTA/100 mm NaCl/ 0.5% NP‐40/1% SDS/1% sodium deoxycholate). The lysates were then subjected to boiling at 100 °C for 15 min, diluted tenfold with NETN buffer containing protease a protease inhibitor cocktail, and centrifuged at 16 000 *g* for 10 min at 4 °C. The resulting supernatants were incubated with appropriate antibodies (for immunoprecipitation) at 4 °C under agitation overnight, and then with protein A/G agarose beads for an additional 3 h at 4 °C on a rotator. Subsequently, the immune‐precipitates were washed with NETN buffer containing 200 mm NaCl, eluted in 2×SDS loading buffer, and the ubiquitination levels were analyzed via western blotting.

### In Vitro Ubiquitination Assay

For in vitro ubiquitination of RFWD3, recombinant proteins, including GST‐RFWD3, GST‐RFWD3^C315A^, His‐UHRF1, His‐UHRF1^5A^, His‐UHRF1^NR2A^, His‐RAD51, His‐RAD51^R254Q^ were expressed in *E.coli* BL21(DE3) and purified as described above. The ubiquitination reaction was performed in a 40 µL reaction system containing 50mm Tris (pH 7.5), 2 mm MgCl_2_, 2mm ATP, and 1mm DTT. The reaction mixture included 100 ng of E1 enzyme (UBA1, Abnova, USA), 300 ng of E2 enzyme (UBC13/MMS2, Abnova, USA), 1 µg of HA‐ubiquitin (R&D Systems, USA), 1 µg of E3 ligase (His‐UHRF1, His‐UHRF1^5A^, or His‐UHRF1^5A^), and 4 µg of substrate (GST‐RFWD3^C315A^). the reaction was incubated at 37 °C for 60 min. To evaluate the inhibitory effects of RAD51 on RFWD3 ubiquitination, 4 µg of His‐RAD51 or His‐RAD51^R254Q^ were added to the reaction system. The reactions were terminated by boiling in 5× SDS‐PAGE loading buffer. Ubiquitination levels were subsequently analyzed by Western blotting.

For in vitro ubiquitination of RAD51, the resin‐bound GST‐RAD51 (≈5 µg) was used in the in vitro ubiquitination reaction. The reaction was carried out as for RFWD3 above.

### HR Reporter Assay

U2OS DR‐GFP cells were seeded at a density of 2 × 10^5^ cells per well in 6‐well plates and subsequently transfected with siRNA with Lipofectamine RNAiMAX (Invitrogen, USA) in accordance with the manufacturer's protocol. Following a 24‐h incubation period, the cells were transfected the second time with 2 µg of an I‐SceI‐expressing plasmid, either alone or with 1 µg of plasmids expressing siRNA‐resistant UHRF1, UHRF1^5A^, UHRF1^NR2A^, or mCherry, through the use of Lipofectamine 3000 (Invitrogen, USA). 48 h after the transfection of I‐SceI, the cells were collected, and the proportion of GFP‐positive cells was determined via flow cytometry. mCherry was used to normalize transfection efficiency. A minimum of 1 x 10⁴ cells were analyzed per sample. The data are expressed as the mean ± S.D. from three independent experiments.

### Statistical Analyses

All statistical analyses were performed with GraphPad Prism (v.9.0) and ImageJ. Data are expressed as mean ± SD. Group comparisons were made using an unpaired two‐sided Student's *t*‐test. *p* < 0.05 was considered statistically significant.

## Conflict of Interest

The authors declare no conflict of interest.

## Author Contributions

G.L. and K.H. contributed equally and co‐first author to this work. G.L. conceived and performed the experiments; K.H., S.L., and Z.S. performed the experiments; G.L. and P.Z. supervised the study and drafted the manuscript.

## Supporting information



Supporting Information

## Data Availability

Data sharing is not applicable to this article as no new data were created or analyzed in this study.

## References

[1] M. Bostick , J. K. Kim , P. O. Esteve , A. Clark , S. Pradhan , S. E. Jacobsen , Science 2007, 317, 1760. 17673620 10.1126/science.1147939

[2] J. Sharif , M. Muto , S.‐I. Takebayashi , I. Suetake , A. Iwamatsu , T. A. Endo , J. Shinga , Y. Mizutani‐Koseki , T. Toyoda , K. Okamura , S. Tajima , K. Mitsuya , M. Okano , H. Koseki , Nature 2007, 450, 908. 17994007 10.1038/nature06397

[3] L. Gao , X. F. Tan , S. Zhang , T. Wu , Z. M. Zhang , H. W. Ai , J. Song , Structure 2018, 26, 304. 29395786 10.1016/j.str.2017.12.016 PMC5803408

[4] S. B. Rothbart , B. M. Dickson , M. S. Ong , K. Krajewski , S. Houliston , D. B. Kireev , C. H. Arrowsmith , B. D. Strahl , Genes Dev. 2013, 27, 1288. 23752590 10.1101/gad.220467.113 PMC3690401

[5] J. Cheng , Y. Yang , J. Fang , J. Xiao , T. Zhu , F. Chen , P. Wang , Z. Li , H. Yang , Y. Xu , J. Biol. Chem. 2013, 288, 1329. 23161542 10.1074/jbc.M112.415398 PMC3543016

[6] S. Xie , J. Jakoncic , C. Qian , J. Mol. Biol. 2012, 415, 318. 22100450 10.1016/j.jmb.2011.11.012

[7] K. Arita , S. Isogai , T. Oda , M. Unoki , K. Sugita , N. Sekiyama , K. Kuwata , R. Hamamoto , H. Tochio , M. Sato , M. Ariyoshi , M. Shirakawa , Proc. Natl. Acad. Sci. USA 2012, 109, 12950. 22837395 10.1073/pnas.1203701109 PMC3420164

[8] N. Nady , A. Lemak , J. R. Walker , G. V. Avvakumov , M. S. Kareta , M. Achour , S. Xue , S. Duan , A. Allali‐Hassani , X. Zuo , Y.‐X. Wang , C. Bronner , F. Chédin , C. H. Arrowsmith , S. Dhe‐Paganon , J. Biol. Chem. 2011, 286, 24300. 21489993 10.1074/jbc.M111.234104 PMC3129210

[9] A. Rottach , C. Frauer , G. Pichler , I. M. Bonapace , F. Spada , H. Leonhardt , Nucleic Acids Res. 2010, 38, 1796. 20026581 10.1093/nar/gkp1152 PMC2847221

[10] P. Karagianni , L. Amazit , J. Qin , J. Wong , Mol. Cell. Biol. 2008, 28, 705. 17967883 10.1128/MCB.01598-07 PMC2223417

[11] E. Rajakumara , Z. Wang , H. Ma , L. Hu , H. Chen , Y. Lin , R. Guo , F. Wu , H. Li , F. Lan , Y. G. Shi , Y. Xu , D. J. Patel , Y. Shi , Mol. Cell 2011, 43, 275. 21777816 10.1016/j.molcel.2011.07.006 PMC4691841

[12] N. Lallous , P. Legrand , A. G. McEwen , S. Ramon‐Maiques , J. P. Samama , C. Birck , PLoS One 2011, 6, 27599. 10.1371/journal.pone.0027599 PMC3214078 22096602

[13] H. Hashimoto , J. R. Horton , X. Zhang , M. Bostick , S. E. Jacobsen , X. Cheng , Nature 2008, 455, 826. 18772888 10.1038/nature07280 PMC2602803

[14] G. V. Avvakumov , J. R. Walker , S. Xue , Y. Li , S. Duan , C. Bronner , C. H. Arrowsmith , S. Dhe‐Paganon , Nature 2008, 455, 822. 18772889 10.1038/nature07273

[15] K. Arita , M. Ariyoshi , H. Tochio , Y. Nakamura , M. Shirakawa , Nature 2008, 455, 818. 18772891 10.1038/nature07249

[16] A. Nishiyama , L. Yamaguchi , J. Sharif , Y. Johmura , T. Kawamura , K. Nakanishi , S. Shimamura , K. Arita , T. Kodama , F. Ishikawa , H. Koseki , M. Nakanishi , Nature 2013, 502, 249. 24013172 10.1038/nature12488

[17] P. A. DaRosa , J. S. Harrison , A. Zelter , T. N. Davis , P. Brzovic , B. Kuhlman , R. E. Klevit , Mol. Cell 2018, 72, 753. 30392931 10.1016/j.molcel.2018.09.029 PMC6239910

[18] M. Mancini , E. Magnani , F. Macchi , I. M. Bonapace , Nucleic Acids Res. 2021, 49, 6053. 33939809 10.1093/nar/gkab293 PMC8216287

[19] C. C. Liang , B. Zhan , Y. Yoshikawa , W. Haas , S. P. Gygi , M. A. Cohn , Cell Rep. 2015, 10, 1947. 25801034 10.1016/j.celrep.2015.02.053 PMC4386029

[20] Y. Tian , M. Paramasivam , G. Ghosal , D. Chen , X. Shen , Y. Huang , S. Akhter , R. Legerski , J. Chen , M. M. Seidman , J. Qin , L. Li , Cell Rep. 2015, 10, 1957. 25818288 10.1016/j.celrep.2015.03.038 PMC4748712

[21] M. K. Ayrapetov , O. Gursoy‐Yuzugullu , C. Xu , Y. Xu , B. D. Price , Proc. Natl. Acad. Sci. USA 2014, 111, 9169. 24927542 10.1073/pnas.1403565111 PMC4078803

[22] H. Zhang , H. Liu , Y. Chen , X. Yang , P. Wang , T. Liu , M. Deng , B. Qin , C. Correia , S. Lee , J. Kim , M. Sparks , A. A. Nair , D. L. Evans , K. R. Kalari , P. Zhang , L. Wang , Z. You , S. H. Kaufmann , Z. Lou , H. Pei , Nat. Commun. 2016, 7, 10201. 26727879 10.1038/ncomms10201 PMC4728409

[23] Z. Deng , C. Long , S. Han , Z. Xu , T. Hou , W. Li , X. Wang , X. Liu , J. Biol. Chem. 2024, 300, 107823. 39341501 10.1016/j.jbc.2024.107823 PMC11530599

[24] J. Y. Hahm , J. Y. Kang , J. W. Park , H. Jung , S. B. Seo , BMB Rep. 2020, 53, 112. 31964471 10.5483/BMBRep.2020.53.2.264 PMC7061213

[25] I. E. Wassing , F. Esashi , Semin. Cell Dev. Biol. 2021, 113, 38. 32938550 10.1016/j.semcdb.2020.08.010 PMC8082279

[26] X. Li , W. D. Heyer , Cell Res. 2008, 18, 99. 18166982 10.1038/cr.2008.1 PMC3087377

[27] S. Inano , K. Sato , Y. Katsuki , W. Kobayashi , H. Tanaka , K. Nakajima , S. Nakada , H. Miyoshi , K. Knies , A. Takaori‐Kondo , D. Schindler , M. Ishiai , H. Kurumizaka , M. Takata , Mol. Cell 2017, 66, 622. 28575658 10.1016/j.molcel.2017.04.022

[28] G. Liu , K. Huang , S. Liu , Y. Xie , J. Huang , T. Liang , P. Zhang , Proc. Natl. Acad. Sci. USA 2024, 121, 2410119121. 10.1073/pnas.2410119121 PMC11648659 39621902

[29] Y. Jenkins , V. Markovtsov , W. Lang , P. Sharma , D. Pearsall , J. Warner , C. Franci , B. Huang , J. Huang , G. C. Yam , J. P. Vistan , E. Pali , J. Vialard , M. Janicot , J. B. Lorens , D. G. Payan , Y. Hitoshi , Mol. Biol. Cell 2005, 16, 5621. 16195352 10.1091/mbc.E05-03-0194 PMC1289407

[30] K. M. Ahmed , R. K. Pandita , D. K. Singh , C. R. Hunt , T. K. Pandita , Mol. Cell. Biol. 2018, 38. 10.1128/MCB.00672-17. PMC5902589 29463647

[31] K. Luo , L. Li , Y. Li , C. Wu , Y. Yin , Y. Chen , M. Deng , S. Nowsheen , J. Yuan , Z. Lou , Genes Dev. 2016, 30, 2581. 27941124 10.1101/gad.289439.116 PMC5204351

[32] H. Ma , H. Chen , X. Guo , Z. Wang , M. E. Sowa , L. Zheng , S. Hu , P. Zeng , R. Guo , J. Diao , F. Lan , J. W. Harper , Y. G. Shi , Y. Xu , Y. Shi , Proc. Natl. Acad. Sci. USA 2012, 109, 4828. 22411829 10.1073/pnas.1116349109 PMC3323953

[33] J. Chu , E. A. Loughlin , N. A. Gaur , S. SenBanerjee , V. Jacob , C. Monson , B. Kent , A. Oranu , Y. Ding , C. Ukomadu , K. C. Sadler , Mol. Biol. Cell 2012, 23, 59. 22072796 10.1091/mbc.E11-06-0487 PMC3248904

[34] C. R. Coxon , E. Anscombe , S. J. Harnor , M. P. Martin , B. Carbain , B. T. Golding , I. R. Hardcastle , L. K. Harlow , S. Korolchuk , C. J. Matheson , D. R. Newell , M. E. M. Noble , M. Sivaprakasam , S. J. Tudhope , D. M. Turner , L. Z. Wang , S. R. Wedge , C. Wong , R. J. Griffin , J. A. Endicott , C. Cano , J. Med. Chem. 2017, 60, 1746. 28005359 10.1021/acs.jmedchem.6b01254 PMC6111440

[35] D. J. Junk , R. Cipriano , M. Stampfer , M. W. Jackson , PLoS One 2013, 8, 53776. 10.1371/journal.pone.0053776 PMC3563539 23390492

[36] Y. Ueki , T. Kruse , M. B. Weisser , G. N. Sundell , M. S. Y. Larsen , B. L. Mendez , N. P. Jenkins , D. H. Garvanska , L. Cressey , G. Zhang , N. Davey , G. Montoya , Y. Ivarsson , A. N. Kettenbach , J. Nilsson , Mol. Cell 2019, 76, 953. 31585692 10.1016/j.molcel.2019.08.029 PMC6981294

[37] C. J. Hastie , P. T. Cohen , FEBS Lett. 1998, 431, 357. 9714542 10.1016/s0014-5793(98)00775-3

[38] S. Liu , J. Chu , N. Yucer , M. Leng , S. Y. Wang , B. P. Chen , W. N. Hittelman , Y. Wang , J. Biol. Chem. 2011, 286, 22314. 21558276 10.1074/jbc.M111.222802 PMC3121378

[39] K. Nakanishi , F. Cavallo , E. Brunet , M. Jasin , Methods Mol. Biol. 2011, 745, 283. 21660700 10.1007/978-1-61779-129-1_16 PMC3261721

[40] M. Muto , Y. Kanari , E. Kubo , T. Takabe , T. Kurihara , A. Fujimori , K. Tatsumi , J. Biol. Chem. 2002, 277, 34549. 12084726 10.1074/jbc.M205189200

[41] Q. Liu , J. Gao , C. Zhao , Y. Guo , S. Wang , F. Shen , X. Xing , Y. Luo , DNA Repair (Amst) 2020, 85, 102702. 31731257 10.1016/j.dnarep.2019.102702

[42] A. Satyanarayana , P. Kaldis , Cell Div. 2009, 4, 9. 19445729 10.1186/1747-1028-4-9 PMC2690586

[43] L. Wohlbold , R. P. Fisher , DNA Repair (Amst) 2009, 8, 1018. 19464967 10.1016/j.dnarep.2009.04.009 PMC2725215

[44] A. Satyanarayana , M. B. Hilton , P. Kaldis , Mol. Biol. Cell 2008, 19, 65. 17942597 10.1091/mbc.E07-06-0525 PMC2174178

[45] A. J. Deans , K. K. Khanna , C. J. McNees , C. Mercurio , J. Heierhorst , G. A. McArthur , Cancer Res. 2006, 66, 8219. 16912201 10.1158/0008-5472.CAN-05-3945

[46] D. E. Pefani , R. Latusek , I. Pires , A. M. Grawenda , K. S. Yee , G. Hamilton , L. van der Weyden , F. Esashi , E. M. Hammond , E. O'Neill , Nat. Cell Biol. 2014, 16, 962. 25218637 10.1038/ncb3035 PMC4861244

[47] J. S. Myers , R. Zhao , X. Xu , A. J. Ham , D. Cortez , Cancer Res. 2007, 67, 6685. 17638878 10.1158/0008-5472.CAN-07-0495 PMC2728292

[48] A. H. Walsh , A. Cheng , R. E. Honkanen , FEBS Lett. 1997, 416, 230. 9373158 10.1016/s0014-5793(97)01210-6

