# Supplementary material for: UHRF1 Controls the Timing of RAD51 Removal During DNA Damage Repair Through Suppressing RFWD3
Source: Adv Sci (Weinh). 2025 Sep 12;12(45):e09901. doi: 10.1002/advs.202509901 (PMC12677587; doi:10.1002/advs.202509901)
Supplement: Supplementary file 1 — Supporting Information [file ADVS-12-e09901-s001.docx]

**Figure S1**

**Figure S1. UHRF1 regulates RAD51 expression via controlling RFWD3. A.** Western blotting analysis of RAD51 protein levels. U2OS cells were depleted of *UHRF1* expression with two siRNAs, and RAD51 protein and mRNA levels were analyzed with western blotting or qPCR. **B.** Western blotting analysis of RAD51 protein levels in U2OS cells overexpressing FLAG-tagged *UHRF1*. **C.** Western blotting analysis of RING1, UCHL3, integrin-β1 and RFWD3 protein levels in U2OS cells depleted of *UHRF1*. **D.** Western blotting analysis of RAD51 and UHRF1 protein levels *RFWD3-*overexpressing U2OS cells. **E.** *In vitro* ubiquitination of RAD51.

**Figure S2**

**Figure S2. UHRF1 promotes RFWD3 ubiquitination. A.** Determination of the ubiquitin chain type on RFWD3. HEK293T cells were transfected with plasmids expressing FLAG-RFWD3 and HA-tagged one lysine-containing ubiquitin. FLAG-RFWD3 was immunoprecipitated and blotted for HA and FLAG. **B.** Top panel: GST pulldown assay using GST or GST-RFWD3 to precipitate his-UHRF1. His-UHRF1 was detected with western blotting (the upper panel). The lower panel shows Coomassie blue staining of the purified proteins used in the assay. Bottom panel: GST pulldown assay using GST-RFWD3 to precipitate truncated UHRF1. The pulldown products were analyzed on SDS-PAGE followed by Coomassie blue staining. **C.** Immunoprecipitation assay of the ectopically expressed RFWD3-HA, FLAG-UHRF1 and FLAG-UHRF1^1-780^ in HEK293T cells. **D.** Immunoprecipitation assay of the ectopically expressed *FLAG-UHRF1, RFWD3-HA* and *RFWD3^N791A/R793A^-HA*, *RFWD3^N791A/G792A/R793A^-HA,* or *RFWD3^R793A^-HA* in HEK293T cells. **E.** GST pull down assay using GST-RFWD3 or GST to precipitate his-UHRF1 or his-UHRF1^NR2A^. The pulldown products were analyzed on SDS-PAGE followed by Coomassie blue staining. **F.** Western blotting analysis of RAD51 and RFWD3 protein levels in U2OS cells with *UHRF1* depleted and re-expression of *UHRF1* or *UHRF1^NR2A^*. The cells were treated with cycloheximide (CHX, 50 μg/ml) for the indicated times. **G.** SDS-gel electrophoresis analysis of purified recombinant proteins. The gels were Coomassie blue stained. **H-I.** In vitro ubiquitination of RFWD3. **J.** Western blotting analysis of UHRF1 protein levels in U2OS cells overexpressing *RFWD3*. **K.** Western blotting analysis of RFWD3 protein levels in in U2OS cells overexpressing *UHRF1*.

**Figure S3**

**Figure S3. RAD51 controls RFWD3 protein stability through its interaction with UHRF1. A.** Western blotting analysis of RFWD3 protein levels in U2OS cells depleted of *RAD51* expression with two siRNAs. The cells were treated with cycloheximide (CHX, 50 μg/ml) for the indicated times. **B.** Western blotting analysis of RFWD3 protein levels in U2OS cells depleted of *RAD51* expression with two siRNAs. The cells were treated with cycloheximide (CHX, 50 μg/ml) alone or together with MG132 for the indicated times. **C.** Western blotting analysis RAD51 and RFWD3 protein levels in U2OS cells with *UHRF1* depleted and re-expression of *UHRF1* or *UHRF1^5A^*. The cells were treated with cycloheximide (CHX, 50 μg/ml) for the indicated times.

**Figure S4**

**Figure S4. Analysis of UHRF1 E3 activity. A.** SDS-gel electrophoresis analysis of purified UHRF1. The gel was Coomassie blue stained. **B.** UHRF1 auto-ubiquitination. **C.** Ubiquitination of RFWD3.

**Figure S5**

**Figure S5. PP4 mediates UHRF1 dephosphorylation. A.** Western blotting analysis of RFWD3 protein levels in *UHRF1* knockdown U2OS cells with or without supplementing with exogenous FLAG-tagged *UHRF1*. The cells were also treated with or without 4 µM CDK2i. **B.** Western blotting analysis of RFWD3 protein levels in U2OS cells treated cycloheximide (CHX, 50 μg/ml) and with or without 4 µM CDK2i. **C.** Western blotting analysis of RFWD3 protein levels in U2OS cells expressing CCND1-CDK2 for 24h, followed by treatment with 4 µM CDK2i or DMSO for 4 h. **D.** Western blotting analysis of RFWD3 protein levels in U2OS cells depleted of *CDC14A*, *CDC14B* or *PP4C*. **E.** Analysis of UHRF1 phosphorylation in HEK293T cells depleted of *PP4C* expression. The endogenous UHRF1 was immunoprecipitated from the cells and incubated with or without lamda phosphatase, and blotted with phospho-Ser/Thr antibodies. **F.** Immunoprecipitation analysis of the interaction between ectopically expressed RFWD3-HA and FLAG-PP4R3A in HEK293T cells. **G.** Immunoprecipitation analysis of the interaction between endogenous RFWD3 and ectopically expressed FLAG-UHRF1 or FLAG-UHRF1^FP2A^ in HEK293T cells. **H.** Western blotting analysis of RFWD3 protein levels in U2OS cells. The cells were treated with 4 µM CDK2i or 6 µM PP4 as indicated. **I.** Analysis of UHRF1 phosphorylation in HEK293T cells. The cells were treated with 4 µM CDK2i or 6 µM PP4i for 4 h.

**Figure S6**

**Figure S6. UHRF1 protects RAD51 at DNA damage site. A.** Representative immunofluorescence (IF) images of γH2AX and RFWD3 in U2OS cells treated with 1 µM MMC for 1 h and released into fresh culture media. The cells were harvested at the indicated time of release and processed for immunofluorescent staining for RFWD3 and γH2AX. **B.** Western blotting analysis of RFWD3 protein levels in U2OS cells depleted of *RFWD3,* or *UHRF1* and re-expressing FLAG-tagged *UHRF1*, *UHRF1^5A^*, or *UHRF1^NR2A^*. **C.** Representative images of RAD51 foci. U2OS cells were treated with 1 µM MMC for 1 h and released into fresh culture media containing 4 µM CDK2i for the indicated time and harvested for immunofluorescent staining. The number of RAD51 foci per cell was counted and quantified from 100 cells. **D.** Representative images of RAD51 foci. U2OS cells were treated with 1 µM MMC for 1 h and released into fresh culture media containing 6 µM PP4i for the indicated time and harvested for immunofluorescent staining. The number of RAD51 foci per cell was counted and quantified from 100 cells. **E.** Western blotting analysis of RFWD3 protein levels in U2OS cells depleted of *RFWD3,* or *UHRF1* and re-expressing *UHRF1*, *UHRF1^5A^*, or *UHRF1^NR2A^*. **F.** Representative IF images of RFWD3 foci. U2OS cells depleted *UHRF1* and re-expressing FLAG-tagged *UHRF1*, *UHRF1^5A^*, or *UHRF1^NR2A^* (**E**) were treated with 2 mM HU (hydroxyurea) for 24 h and released into fresh culture media. The cells were harvested at the indicated time of release and processed for immunofluorescent staining for RFWD3. The number of RFWD3 foci/cell were counted and quantified from FLAG IF-positive 100 cells. **G.** Quantification of the number of the indicated foci in (**F**). **H.** Western blotting analysis of protein levels as indicated in U2OS cells for DR-GFP assays. U2OS cells were depleted *UHRF1* and re-expressing FLAG-tagged *UHRF1*, *UHRF1^5A^*, or *UHRF1^NR2A^*. Data (mean ± S.D.) from 3 independent experiments are presented. * Indicates p <0.05, ** p <0.01, *** p <0.001 and **** p <0.0001. Scale bar, 5 µm.

**Reagents used in the study**

| **REAGENT or RESOURCE** | **SOURCE** | **IDENTIFIER** |
| --- | --- | --- |
| **Antibodies** |  |  |
| anti-FLAG Antibody (WB: 1:3000) | Sigma | F1804 |
| anti-HA Antibody (WB: 1:3000) | Sigma | H6908 |
| RFWD3 antibody(IP: 1:100; WB: 1:1000) | GeneTex | GTX116292 |
| RFWD3 antibody(IF:1:200) | Fine test | FNab07257 |
| UHRF1 Antibody (WB: 1:1000; IP: 1:200) | GeneTex | GTX113963 |
| RAD51 Antibody (IF: 1:1000) | Abcam | ab176458 |
| RPA1 antibody (WB:1:1000; IF:1:500) | Cell signaling | 2267s |
| RAD51 Antibody (WB: 1:1000) | ABclonal | A2829 |
| RING1 (WB: 1:1000) | ABclonal | A20171 |
| UCHL3 (WB: 1:1000) | ABclonal | A1372 |
| PP4C(WB: 1:1000) | ABclonal | A13531 |
| PP4R3A(WB: 1:1000) | ABclonal | A8500 |
| Integrin-beta-1 (WB: 1:1000) | ABclonal | A27421 |
| β-tubulin Antibody (WB: 1:10000) | ABclonal | A12289 |
| GAPDH Antibody (WB: 1:10000) | Proteintech | 60004-1-Ig |
| anti-His Antibody (WB: 1:5000) | Proteintech | 66005-1-Ig |
| γH2A Antibody (IF: 1:1000) | Abcam | ab81299 |
| ubiquitin Antibody (WB: 1:1000) | Cell signaling | #70990 |
| ubiquitin Antibody (WB: 1:1000) | Proteintech | 10201-2-AP |
| Lamin B Antibody (WB: 1:10000) | Proteintech | 12987-1-AP |
| Normal Rabbit IgG | Cell signaling | #2729 |
| Peroxidase-AffiniPure Goat Anti-Mouse IgG（H+L）(WB: 1:5000) | Jackson | 115-035-003 |
| Peroxidase-AffiniPure Goat Anti-Rabbit IgG（H+L）(WB: 1:5000) | Jackson | 111-035-003 |
| Goat anti-Mouse lgG (H+L) Highly Cross-Adsorbed Secondary Antibody, Alexa FluorTm 568 (IF: 1:1000) | Thermo | A-11031 |
| Goat anti-Rabbit IgG (H+L) Highly Cross-Adsorbed Secondary Antibody, Alexa Fluor™ 488 (IF: 1:1000) | Thermo | A-11034 |
| Goat anti-Rabbit IgG (H+L) Highly Cross-Adsorbed Secondary Antibody, Alexa Fluor™ 568 (IF: 1:1000) | Thermo | A-11036 |
| Goat anti-Mouse IgG (H+L) Highly Cross-Adsorbed Secondary Antibody, Alexa Fluor™ 488 (IF: 1:1000) | Thermo | A-11029 |
| **Chemicals, Peptides, and reconmbinant proteins** |  |  |
| CDK2-IN-4 | MCE | HY-117535 |
| fostriecin sodium salt (PP4i) | Medbio | MED11723 |
| Doxycycline | Doxycycline | D9891 |
| λ phosphatase | MCE | HY-E70373 |
| **Recombinat DNA** |  |  |
| pET30a | Addgene | #85761 |
| pGEX-6P-1 | Amersham | HG-VYA0225 |
| pET30a-his-UHRF1 | This paper | N/A |
| pET30a-his-UHRF1-1-724 | This paper | N/A |
| pET30a-his-UHRF1-1-745 | This paper | N/A |
| pET30a-his-UHRF1-1-750 | This paper | N/A |
| pET30a-his-UHRF1-1-753 | This paper | N/A |
| pET30a-his-UHRF1-1-755 | This paper | N/A |
| pET30a-his-UHRF1-NR2A | This paper | N/A |
| pET30a-his-UHRF1-5A | This paper | N/A |
| pET30a-his-RAD51 | This paper | N/A |
| pET30a-his-RAD51-R254Q | This paper | N/A |
| pGEX-6P-1-GST-RFWD3 | This paper | N/A |
| pGEX-6P-1-GST-UHRF1 | This paper | N/A |
| pCMV-HA | MIAOLING BIOLOGY | P0818 |
| pCDH-EF1-FLAG | MIAOLING BIOLOGY | P22492 |
| pInducer20 | BioVector NTCC | 44012 |
| pCMV-HA-RAD51 | This paper | N/A |
| pCMV-HA-RAD51-R254Q | This paper | N/A |
| pInducer20-HA-RAD51 | This paper | N/A |
| pInducer20-HA-RAD51-R254Q | This paper | N/A |
| pInducer20-HA-RAD51-D231A | This paper | N/A |
| pCDH-EF1-FLAG-RAD51 | This paper | N/A |
| pCMV-HA-UHRF1 | This paper | N/A |
| pCMV-HA-CCND1-CDK2 |  |  |
| pCMV-HA-UHRF1-CH2A | This paper | N/A |
| pCMV-HA-UHRF1-NR2A | This paper | N/A |
| pCMV-HA-UHRF1-5A | This paper | N/A |
| pCMV-HA-UHRF1-S661A | This paper | N/A |
| pCMV-HA-UHRF1-S661D | This paper | N/A |
| pCMV-HA-UHRF1-FP2A | This paper | N/A |
| pCMV-HA-UHRF1-F757AP760A | This paper | N/A |
| pCMV-HA-RFWD3 | This paper | N/A |
| pCDH-EF1-FLAG-UHRF1-NR2A | This paper | N/A |
| pCDH-EF1-FLAG-UHRF1-CH2A | This paper | N/A |
| pCDH-EF1-FLAG-UHRF1-5A | This paper | N/A |
| pCDH-EF1-FLAG-UHRF1-S661A | This paper | N/A |
| pCDH-EF1-FLAG-UHRF1-S661D | This paper | N/A |
| pCDH-EF1-FLAG-UHRF1 | This paper | N/A |
| pCDH-EF1-FLAG-UHRF1-1-780 | This paper | N/A |
| pCDH-EF1-FLAG-PP4R3A | This paper | N/A |
| pCDH-EF1-FLAG-UHRF1-1-FP2A | This paper | N/A |
| pCDH-EF1-FLAG-UHRF1-F757AP760A | This paper | N/A |
| pInducer20-GFP-RAD51 | This paper | N/A |
| pInducer20-GFP-RAD51-R254Q | This paper | N/A |
| **Software and algorithms** | This paper | N/A |
| ImageJ | <https://imagej.net/ij/index.html> | N/A |
| Graphpad Prism | GraphPad Software | N/A |
| **Oligonucleotides** |  |  |
| siCtrl:5'-CGUACGCGGAAUACUUCGA-3' | GenePharma | N/A |
| RAD51 si#1 : AAGGGAAUUAGUGAAGCCAAA | GenePharma | N/A |
| RAD51 sh#1 : AAGGGAATTAGTGAAGCCAAA | GenePharma | N/A |
| RAD51 si#2: CAGGUGGUAGCUCAAGUGGAU | GenePharma | N/A |
| RAD51 sh#2: CAGGTGGTAGCTCAAGTGGAT | GenePharma | N/A |
| UHRF1 si#1: GCCUUUGAUUCGUUCCUUCUU | GenePharma | N/A |
| UHRF1 si#2: GCGCUGGCUCUCAACUGCUUU | GenePharma | N/A |
| RFWD3 si#1: AGGAUGUGAGUGGGCAUCAAGCAUU | GenePharma | N/A |
| RFWD3 si#2: GGACCUACUUGCAAACUAUtt | GenePharma | N/A |
| PP4C si#1: GACAAUCGACCGAAAGCAAdTdT | GenePharma | N/A |
| PP4C si#2: UUGCUUUCGGUCGAUUGUCdCdG | GenePharma | N/A |
| PPR4R3A#1 : CGTCATTGGATGTTTAGAATA | GenePharma | N/A |
| PPR4R3A#1 : GCACTTGTATTGGAATTGTTA | GenePharma | N/A |
| qPCRr-GAPDH-F: GTCTCCTCTGACTTCAACAGCG | TsingKe | N/A |
| qPCR-GAPDH-R: ACCACCCTGTTGCTGTAGCCAA | TsingKe | N/A |
| qPCR-RAD51-F: GGAAGAAGCTGGATTCCATACTG | TsingKe | N/A |
| qPCR-RAD51-R: GATCTCTGACCGCCTTTGGTG | TsingKe | N/A |
| UHRF1-FLAG-F: GGATGACGATGACAAGCTTGGTACCATGTGGATCCAGGTTCGGACCATGGACG | TsingKe | N/A |
| UHRF1-FLAG-R: CTTCCTCTGCCCTCAGCGGCCGCGGATCCCCGGCCATTGCCGTAGC | TsingKe | N/A |
| UHRF1-HA-F: TTGGTACCGAGCTCGGATCCGCCACCATGTGGATCCAGGTTCGGAC | TsingKe | N/A |
| UHRF1-HA-R: CATAATCAGGTACGTCGTATGGGTACTCGAGCCGGCCATTGCCGTAGC | TsingKe | N/A |
| RAD51-HA-F: TTGGTACCGAGCTCGGATCCGCCACCATGGCAATGCAGATGCAGC | TsingKe | N/A |
| RAD51-HA-R: CATAATCAGGTACGTCGTATGGGTACTCGAGTCAGTCTTTGGCATCTCCCACTC | TsingKe | N/A |
| UHRF1-GST-F: GTTCCAGGGGCCCCTGGGATCCATGGACGGGAGGCAGACC | TsingKe | N/A |
| UHRF1-GST-R: CAGTCACGATGCGGCCGCTCGAGCCGGCCATTGCCGTAGCC | TsingKe | N/A |
| RAD51-His-F: CCATGGCTGATATCGGATCCATGGCAATGCAGATGCAGC | TsingKe | N/A |
| RAD51-His-R: TGGTGGTGGTGGTGCTCGAGTCATCAGTCTTTGGCATCTCCCAC | TsingKe | N/A |
| RAD51-GST-F: GTTCCAGGGGCCCCTGGGATCCATGGCAATGCAGATGCAGC | TsingKe | N/A |
| RAD51-R254Q-F: TTTCTGCGGATGCTTCTGCAACTCGCTGATGAGTTTGGTGTA | TsingKe | N/A |
| RAD51-R254Q-R: TACACCAAACTCATCAGCGAGTTGCAGAAGCATCCGCAGAAA | TsingKe | N/A |
| pInducer-GFP-RAD51-F: TAGTGAACCGTCAGATCGCCTGGAGACGATGGTGAGCAAGGGCGAG | TsingKe | N/A |
| pInducer-GFP-RAD51-R: TAGGCCTCTAGACAATTGGTCGACCTAGTCTTTGGCATCTCCCACTCCA | TsingKe | N/A |
| UHRF1-His-F: CCATGGCTGATATCGGATCCATGTGGATCCAGGTTCGGAC | TsingKe | N/A |
| UHRF1-1-724-R: TGGTGGTGGTGGTGCTCGAGTCAacactggaacgtctcctccac | TsingKe | N/A |
| UHRF1-1-745-R: TGGTGGTGGTGGTGCTCGAGTCActtgcacacgttgtgctggca | TsingKe | N/A |
| UHRF1-1-750-R: TGGTGGTGGTGGTGCTCGAGTCAtctgtccaggcagtccttgc | TsingKe | N/A |
| UHRF1-1-753-R: TGGTGGTGGTGGTGCTCGAGTCAccgaaaggatctgtccaggca | TsingKe | N/A |
| UHRF1-1-755-R: TGGTGGTGGTGGTGCTCGAGTCActgtgcccgaaaggatctgtc | TsingKe | N/A |
| UHRF1-His-1-741-R: TGGTGGTGGTGGTGCTCGAGTCAGTGCTGGCACACGGTCG | TsingKe | N/A |
| UHRF1-His-1-751-R: TGGTGGTGGTGGTGCTCGAGTCAGGATCTGTCCAGGCAGTCCTT | TsingKe | N/A |
| UHRF1-His-R: TGGTGGTGGTGGTGCTCGAGTCACCGGCCATTGCCGTAGCC | TsingKe | N/A |
| UHRF1-5A-F: TGCGCGGCCTGCGCGGCCGCATCC | TsingKe | N/A |
| UHRF1-5A--R: GGATGCGGCCGCGCAGGCCGCGCA | TsingKe | N/A |
| Pet30-UH-NR2A-R: TGGTGGTGGTGGTGCTCGAGTCAcgcagcagcagcgtagcc | TsingKe | N/A |
| UHRF1-FASP-F: AGCAGCAGGAGGGGGGCGCCGCGTCCGCCAGGACGGGCAAGGGCAAG | TsingKe | N/A |
| UHRF1-FASP-R: CTTGCCCTTGCCCGTCCTGGCGGACGCGGCGCCCCCCTCCTGCTGCT | TsingKe | N/A |
| UHRF1-FSCP-R1: CTGCAGAGGCTGGTTCACCTGCATGGCATAGCTGCGGCCCAGGTC GTAGCGGCAGGCAGCGCAGCTGGCCACCTGTGCCCGAAAGGAT | TsingKe | N/A |
| UHRF1-FSCP-R2-HA:AATCAGGTACGTCGTATGGGTACTCGAGCCGGCCATTGCCGTAGC CGGGGAAGAGCTGGTTGAGGACGGTCTGCAGAGGCTGGTTCAC | TsingKe | N/A |
| GST-RFWD3-F: TCCAGGGGCCCCTGGGATCCATGGCTCATGAAGCAATGGAATATGATG | TsingKe | N/A |
| GST-RFWD3-R: CACGATGCGGCCGCTCGAGTCACTCCCACTTATAGATGTGGACCAT | TsingKe | N/A |
| FLAG-RFWD3-F: gatgacgatgacaagcttggtaccatgggatccATGGCTCATGAAGCAATGGAATATG | TsingKe | N/A |
| FLAG-RFWD3-R: cttcctctgccctcagcggccgcggatccCTCCCACTTATAGATGTGGACCATC | TsingKe | N/A |
| HA-RFWD3-F: TTGGTACCGAGCTCGGATCCgccaccATGGCTCATGAAGCAATGGAA | TsingKe | N/A |
| HA-RFWD3-R: TACGTCGTATGGGTACTCGAGCTCCCACTTATAGATGTGGACCATC | TsingKe | N/A |
| pcmv-CCND1-F:TTGGTACCGAGCTCGGATCCgccaccatggaacaccagctcctg | TsingKe | N/A |
| mid-link-F:gtgcgggacgtggacatctccgggtccgggtccgggtccgggatggagaacttccaaaaggtggaa | TsingKe | N/A |
| mid-link-R:ttccaccttttggaagttctccatcccggacccggacccggacccggagatgtccacgtcccgcac | TsingKe | N/A |
| pcmv-CDK2-R:TACGTCGTATGGGTACTCGAGgagtcgaagatggggtactgg | TsingKe | N/A |

**Uncropped gels and blots**

**Figure 1**

**Figure 2**

**Figure 3**

**Figure 4**

**Figure 5**

**Figure S1**

**Figure S2**

**Figure S3**

**Figure S4**

**Figure S5**

**Figure S6**
